# Development of a Semi-Mechanistic Modeling Framework for Wet Bead Milling of Pharmaceutical Nanosuspensions

**DOI:** 10.3390/pharmaceutics16030394

**Published:** 2024-03-13

**Authors:** Donald J. Clancy, Gulenay Guner, Sayantan Chattoraj, Helen Yao, M. Connor Faith, Zahra Salahshoor, Kailey N. Martin, Ecevit Bilgili

**Affiliations:** 1 GlaxoSmithKline R&D, Collegeville, PA 19426, USA; gulenay.x.guner@gsk.com (G.G.); sayantan.x.chattoraj@gsk.com (S.C.); helen.f.yao@gsk.com (H.Y.); connor.x.faith@gsk.com (M.C.F.); zahra.x.salahshoor@gsk.com (Z.S.); kailey.x.martin@gsk.com (K.N.M.); 2 Otto H. York Department of Chemical and Materials Engineering, New Jersey Institute of Technology, Newark, NJ 07102, USA; ecevit.bilgili@njit.edu

**Keywords:** milling, wet bead milling, particle size prediction, modeling, semi-mechanistic modeling, microhydrodynamic model, process scale-up, process optimization

## Abstract

This study aimed to develop a practical semi-mechanistic modeling framework to predict particle size evolution during wet bead milling of pharmaceutical nanosuspensions over a wide range of process conditions and milling scales. The model incorporates process parameters, formulation parameters, and equipment-specific parameters such as rotor speed, bead type, bead size, bead loading, active pharmaceutical ingredient (API) mass, temperature, API loading, maximum bead volume, blade diameter, distance between blade and wall, and an efficiency parameter. The characteristic particle size quantiles, i.e., *x*_10_, *x*_50_, and *x*_90_, were transformed to obtain a linear relationship with time, while the general functional form of the apparent breakage rate constant of this relationship was derived based on three models with different complexity levels. Model A, the most complex and general model, was derived directly from microhydrodynamics. Model B is a simpler model based on a power-law function of process parameters. Model C is the simplest model, which is the pre-calibrated version of Model B based on data collected from different mills across scales, formulations, and drug products. Being simple and computationally convenient, Model C is expected to reduce the amount of experimentation needed to develop and optimize the wet bead milling process and streamline scale-up and/or scale-out.

## 1. Introduction

Wet bead milling (also known as wet stirred media milling) is a unit operation used in the pharmaceutical industry for the preparation of suspension-based products [1]. A recent survey indicates that wet bead milling is the preferred approach for the preparation of ultrafine drug suspensions and nanosuspensions compared to other techniques, such as liquid antisolvent precipitation and high-pressure homogenization [2]. This is not unexpected since wet bead milling has several advantages as it is a robust, reproducible, scalable, organic solvent-free, and environmentally friendly process [3,4]. It enables the preparation of concentrated stable suspensions of drug particles [3], which can have several applications in drug delivery such as modulating drug dissolution and absorption [5,6,7,8,9] and the design of long acting injectables (LAI) [10,11,12,13].

Developing the fundamental mechanistic understanding of manufacturing processes, underpinned by a science- and risk-based approach, is a key element of the Quality-by-Design (QbD) framework of product development, aligned with the expectations outlined in the International Council on Harmonization (ICH) guidance Q8 (R2), Q9, and Q10 [14]. In this work, we have focused our efforts on developing an enhanced level of process understanding of wet bead milling to support the development of the predictive design capability for this unit operation based on process modeling. There is a need to enhance the mechanistic understanding of potential process-related challenges encountered during wet bead milling, guided by the QbD principles of development. These failure modes may include poor milling efficiency due to aggregation and Ostwald ripening [15,16,17], long processing times [3,18,19], bead wear and product contamination [20,21], potential chemical degradation [22], and mechanical or thermal-stress-induced solid-state transformations and generation of higher energy metastable phases during milling, such as amorphization and polymorphic transitions [22,23].

The modeling of the wet bead milling process can significantly help process development and optimization [24,25] and offers multiple benefits; at a minimum, mechanistic or first-principle-based models provide a quantitative, fundamental understanding of the impact of operation–design parameters. Since APIs are typically very expensive, there is a need to predict their behavior in the unit operations prior to committing a significant amount of material in the developmental stage. Evaluating the behavior of a formulation in wet bead milling and establishing a design space typically requires significant investment in time and material. Computational models for milling performance would be beneficial not only because they reduce API quantities needed for process development but also because they would help inform product development teams in advance regarding whether a process is likely to achieve reasonable throughputs at production scale.

Interestingly, statistically based models such as empirical regression fits, response surface methodology (RSM), etc., have been overwhelmingly preferred over the mechanistic–phenomenological models according to a review of the wet bead milling modeling in the pharmaceutical nanotechnology literature [25]. This is not surprising: (i) Except the SI–SN [26,27] and the MHD models [28,29], the mechanistic–phenomenological models are computationally expensive, requiring specialized software and expertise. (ii) The usage of the SI–SN and the MHD models can be limited if reliable information on model inputs such as average power consumption during milling, the apparent shear viscosity, and the density of the suspension are not available. (iii) Except for PBM, no complex models such as CFD [30,31] or DEM [32,33,34] consider the evolving PSD due to particle breakage. And (iv) PBMs must incorporate the operation–design parameters into the kernels for process predictions, which makes model calibration difficult and requires computationally intensive global optimizers for parameter estimation. On the other hand, statistically based models are easier to develop and use and are more accessible to pharmaceutical engineers and scientists. Characteristic drug particle sizes (*x*_50_ and *x*_90_) or specific surface area can be described and/or predicted by RSM and regression analysis as a function of formulation and process parameters. Most of the statistically based empirical studies have correlated the particle sizes with the milling time and speed [35,36,37,38]; only a handful of studies have additionally considered bead size [39,40], bead loading [41], or API loading [42]. Other studies have considered only bead loading [43,44] or milling time and bead loading [45,46]. Finally, empirical breakage kinetic models such as the first-order kinetics, *n*^th^-order kinetics, and warped-time model have been used to describe the timewise evolution of the median drug particle size or specific (external) surface area [35,47,48]. An empirical correlation for the breakage rate constant of the first-order kinetic model based on stirrer speed and a scaling factor was developed in [35], but no direct scale-up was demonstrated. Moreover, such empirical correlations are only applicable to the specific process, unless its application is demonstrated for different case studies. The breakage rate constant of the *n*^th^-order model was successfully correlated with the MHD parameters to a lab-scale mill; however, explicit correlation of the MHD parameters on the process parameters has not been established, and scale-up has not been considered [48].

The take-away of the above analysis of the statistically based empirical models is that despite the obvious need for pharmaceutical manufacturing engineers to have a good model for prediction and optimization of wet bead milling processes across scale, incorporating the impacts of all process–formulation–design parameters, it is not easy to develop such a model. Besides being practical and computationally convenient, it should also predict impacts of changes in process parameters and scale-up or scale-out across equipment. One such model [47] described the evolution of the specific surface area using a first-order kinetic model with a characteristic time constant, which was correlated with the rotation speed, bead loading, and API loading. The time constant is purely empirical with no physical (microhydrodynamic) basis, and it does not include the effects of bead properties, which have a significant impact on breakage kinetics, cycle time, and energy consumption [20,29].

The aim of this study is to develop a practical semi-mechanistic modeling framework that is computationally efficient, easy to use, and convenient to calculate and that can be used as an engineering tool during process development. The scope of this work included an analysis of six internal GSK drug products and two drug products at NJIT as milled by six mills across six different scales, with an expected outcome that a common model can be used to model every scale and drug product. A challenge to creating a widely encompassing model for wet bead milling is the high number of potential parameters that are important. Developing a practical model with a large number of independent variables, including process parameters, formulation parameters, and equipment-specific parameter was an ambitious undertaking. To this end, in this work, we developed three models with different levels of complexity and mechanistic rigor. First, we linearized the reciprocal of the characteristic particle size quantiles (*x*_10_, *x*_50_, *x*_90_) to describe the timewise particle size evolution. Then, we used microhydrodynamic theory [20,29] to derive the general functional form of the apparent breakage rate constant, which accounts for the impact of all process parameters and bead properties. The apparent breakage rate constant was then derived as a linear function of the frequency of drug particle compressions between the beads to obtain Model A. As the most general and demanding model, Model A requires one to calculate the granular temperature, which can be estimated by solving the power dissipation equation in the microhydrodynamic model [25]. Model B was developed by assuming a general power-law expression for the granular temperature. This simplification has general applicability and great practical utility as we can obtain the exponents of the process parameters in Model B by fitting it to experimental data. Here, we fit Model B to several case studies conducted by GSK and NJIT on various stirred media mills of multiple scales, which resulted in common exponents for each process parameter, resulting in the simplest model, i.e., Model C. Overall, we have formulated a modeling framework encompassing models with varying complexity and practical utility, and we hope that such models can be adopted and/or adapted by engineers for optimization, scale-out, and scale-up of wet bead milling processes.

## 2. Materials and Methods

### 2.1. Materials

The APIs used for investigation at NJIT were fenofibrate (BP grade, purchased from Jai Radhe Sales Ahmedabad, India) and griseofulvin (BP/EP grade micronized, Letco Medical, Decatur, AL, USA). The drug product formulations at GSK are proprietary APIs that will not be listed explicitly but simply referred to as a drug product (DP) number, i.e., DP1, DP2, … DP8. Both NJIT studies used Hydroxypropyl cellulose (HPC, L grade, Nisso America Inc., New York, NY, USA) as a nonionic polymeric stabilizer, and sodium dodecyl sulfate (SDS, ACS grade, GFS chemicals, Columbus, OH, USA) as an anionic surfactant where the formulation was 10% drug, 7.5% HPC-L and 0.05% SDS with respect to 200 g DI water [49,50]. In addition, 400 μm nominal sized Zirmil Y grade zirconia beads and HCC grade polystyrene beads were purchased from Saint Gobain ZirPro (Mountainside, NJ, USA) and Norstone Inc. (Bridgeport, PA, USA), respectively. Additionally, GSK formulations contained commonly used excipients such as surfactants, stabilizers, etc.

### 2.2. Experimental Setup

Each of the experiments described in this manuscript involved wet bead milling in one or more of six different mills, purchased from Netzsch Fine Particle Size Technology, LLC (Exton, PA, USA). A tabulated list of the relevant mill characteristics and parameter ranges explored is shown in Table 1. The experiments conducted on each mill varied substantially in the process parameter operating space. A total of 64 runs were executed in the mills, respectively, across 8 different drug products.

### 2.3. Particle Size Measurement

For particle size measurements performed at GSK, the PSD of the drug suspension at various milling times was determined using laser diffraction by Malvern Mastersizer 3000 particle size analyzer (software v3.81 or validated equivalent) with Hydro MV Dispersion Unit and Temperature Control Unit. At NJIT, the particle size distribution (PSD) of the drug suspensions at various milling times was determined by LS 13-320 Beckman Coulter instrument (Brea, CA, USA). While the sampling interval varies for each experiment depending on the total milling time at GSK, predefined time intervals (2 s, s = 0, 1, 2, … 7 min) with the addition of 40 s, 24 min, 48 min, 96 min, 128 min and 180 min were used at NJIT.

For measurements performed in GSK, ~2 mL samples of suspension were taken from the bulk holding tank at predefined time intervals. Two drops from each sample were transferred via an 18G needle (or equivalent) to a microcentrifuge tube containing ~1 mL of water for injection (WFI). The sample with WFI dilution was then mixed to homogeneity. During measurements, obscuration was maintained at 4–6%. Measurements were repeated three times, and the average and standard deviation of these measurements were determined. The Malvern instrument was turned on for no less than 30 min prior to use.

For the particle size measurements performed at NJIT, the samples were taken from the mill outlet at predefined time intervals. The final sample was taken from the holding tank and all samples were measured with laser diffraction. Before each measurement, a suspension sample (~2.0 mL for griseofulvin and ~1.0 mL for fenofibrate) was diluted with 5.0 mL of the respective vehicle using a vortex mixer (Fisher Scientific Digital Vortex Mixer, Model No: 945415, Pittsburgh, PA, USA) at 1500 rpm for a minute. During measurements, polarized intensity differential scattering (PIDS) was maintained between 40% and 50%, while the obscuration was maintained below 8%. PSD was provided by the equipment software, which used the Mie scattering theory. The refractive indices of GF, FNB and water were taken as 1.65, 1.55, and 1.33, respectively [49,50]. Measurements were repeated four times, and the average and standard deviation of these measurements were determined.

### 2.4. Young’s Modulus of Compacts

A compaction simulator (Styl’One Evolution, Medelpharm, Beynost, France) was used to produce compacts of each API (with no excipients) with a round flat-faced 11.28 mm B punch at a target compression speed. The compression cycle included a precompression phase followed by main compression. The methodology reported by Mazel et al. [51] was used in this work to measure the Young’s modulus (*YM*) of the bulk API materials based on compaction analysis. To obtain the Young’s modulus as a function of compact porosity, a range of main compression forces were applied to the powders in the die. For each compaction, ~500 mg of API was used. The tablet weight and thickness values were measured, and the out-of-die porosity of each compact was calculated using $\varepsilon =\frac{\frac{m_{c}}{V_{c}}}{\rho }$, where *m*_c_ and *V*_c_ are the weight and volume of the out-of-die compact, and *ρ* is the true density of API powder [51].

For measuring *YM*, the compact must only be undergoing elastic deformation. This corresponds to the linear part of compression pressure–tablet thickness curve. During elastic deformation, the linear stress–strain relationship can be written as ${(\sigma }_{ax}-2PR\sigma_{rad})=YM-YM\frac{h}{h_{0}}$, where *σ*_ax_ and *σ*_rad_ are axial and radial stresses equal to the pressure levels (P_ax_ is a mean value of lower and upper punch pressures, and P_rad_ is the radial pressure to the die wall), *PR* is Poisson’s ratio, *h* is the thickness of the powder bed, and *h*_0_ is the initial thickness. Plotting ${(\sigma }_{ax}-2PR\sigma_{rad})$ vs. *h* provides a linear relationship with *YM* as its intercept, and *YM* can be obtained at different pressure levels that yield different compact porosities [51].

An exponential decay function was fit to *YM* versus porosity data, and the *YM* at the zero compact porosity was estimated for the model compounds in this study.

## 3. Theoretical

The approach to model the timewise particle size evolution during wet bead milling entails first establishing a data transformation to linearize the characteristic particle size quantiles *x*_10_, *x*_50_, and *x*_90_ of the cumulative PSD (Section 3.1) and then developing a model to predict the timewise evolution of linearized particle sizes.

**Figure 1:**
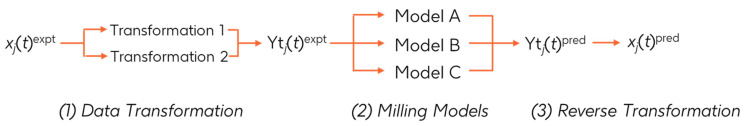
Model training workflow comprising three major steps: (**1**) data transformation to linearize characteristic particle size quantiles *x*_j_(*t*) (*j* = 10, 50, 90) via Transformation 1 (Equation (1)) or Transformation 2 (Equation (2)); (**2**) model fitting to capture particle size evolution during wet bead milling via Model A (Equation (4)), Model B (Equation (5)) or Model C (Equation (6)); (**3**) reverse data transformation to return particle size quantiles.

The general model training workflow is presented in Figure 1.

### 3.1. Particle Size Data Transformation for Linearization

One of the challenges of modeling wet bead milling is the highly nonlinear and potentially sigmoidal nature of the timewise variation in the characteristic particle sizes of *x*_10_, *x*_50_, and *x*_90_. Like most types of models, there is a substantial benefit to simplifying the model by linearizing the input data with respect to time. This section describes the data transformation that we employed for data linearization. While other data transformation strategies may also be useful, we justified the transformation here through two different theoretical approaches in the milling literature and derived them based on breakage kinetics models (Appendix A).

For drug products where the drug crystals tend to exist as individual crystals, we applied model fits to the complete dataset including the initial particle sizes. However, we disregarded a few data points in the neighborhood of time *t* = 0 [52,53] for the drugs that tend to agglomerate, which can be justified by the following considerations. First, the initial specific breakage rate of the coarse particles and any drug clusters (agglomerates) is so high that it, when compared with that of the fine particles and nanoparticles, may not be adequately captured by simple empirical kinetic models, even if they account for the size dependence of the specific breakage rate. If agglomerates are initially present, they are broken down in the mill during the first few turnovers. Here, the number of turnovers *N*_t_ is defined by *N*_t_ = *Qt/V*_tb_, where *Q* is the pumping rate (suspension flow rate) and *V*_tb_ is the total batch volume. Since crystal agglomeration is subject to significant variation even within the same batch from crystallization, these initial particle size data may be subject to noise (see also the discussion in [35]), making these not meaningful for the prediction of the behavior after completion of the first few turnovers. Thus, it follows that initial breakage of coarse particles and deagglomeration of clusters should be omitted since the main focus of wet bead milling process development is the breakage of particles into sub-micron size (i.e., the behavior after the first few turnovers). Also, from a theoretical standpoint, the transform we used is supported by an analytical solution of a self-similar PBM [54] away from the initial condition.

After discarding the particle size data for the first few turnovers, the second step in the data transformation strategy was to take the inverse of *x*_10_, *x*_50_, and *x*_90_. Our experience suggests that this part of the transformation process mostly linearizes the data, though some curvature with respect to time and sigmoidal behavior is expected if the milling process is pushed to near the apparent grinding limit. When the milling process is not nearing the grinding limit and no sigmoidal behavior is observed, then only the third step of the transformation is needed. In this third step (see Equation (1)), a shape factor *N_j_* was introduced for each inverse particle size quantile, which minimizes the error when a line is fitted through the data with respect to time. The proposed transform Yt of the *j*^th^ size quantile, which can be derived by a semi-empirical *n*^th^-order rate-based model (see Appendix A), is expressed as in Equation (1):

(1) $${Yt}_{j}={Yt}_{j}\left(1/x_{j}\right)={\left(1/x_{j}\right)}^{N_{j}}=k_{j}t+B_{j}$$

where *j* = 10, 50 and 90, and *k_j_* is the apparent breakage rate constant that varies with the process conditions. Here, *B_j_* is a parameter that was obtained by fitting to the dynamic data. From a theoretical standpoint, it is related to the initial feed PSD prior to milling. *B_j_* equals either the value of (1/*x_j_*(0))*^N^_j_* of the actual feed PSD at *t* = 0 or its value for a theoretical feed PSD that is self-similar with the asymptotic self-similar size distribution at longer milling times (see the discussion on the self-similar solution of a PBM for milling in Appendix A). However, *B_j_* was ultimately obtained from fitting because it is not expected to satisfy the actual initial condition due to the issues of using the initial time points, as discussed in the previous section. It is assumed that both *N_j_* and *B_j_* are invariant to the processing conditions and that the kinetic influence of all process variation is captured by *k_j_*. Though the model will have higher fidelity if *N_j_* and *B_j_* are separately fitted to the particle size evolution of each batch/experiment with different processing conditions, such a model will have little predictive capability and will not be useful. Hence, the success of our model largely rests upon how well *k_j_* captures the process variations across scales. We note, however, that the fitted values of *N_j_* for each particle size quantile can and likely will be different from each other when fitted separately. Equation (1) is supported by two theoretical approaches which are self-similar solution of PBM and microhydrodynamic model along with Charles’ energy–average particle size relationship (see Appendix A).

Equation (1) and the theoretical approaches suggest *x_j_* → 0 and 1/*x_j_* → ¥ as *t* → ¥, which may not pose a serious problem unless the milling is prolonged to approach an apparent grinding limit size *x*_*j*,inf_ [55]. Thus, if the data for 1/*x_j_* display sigmoidal behavior at long milling times, the best approach is to fit a transform that is similar to the “Prout-Thompkins” transform utilized in auto-catalyzed solid state kinetics [56] as shown in Equation (2):

(2) $${Yt}_{j}={Yt}_{j}\left(1/x_{j}\right)={\left(\frac{1/x_{j}}{1/x_{j,inf}-1/x_{j}}\right)}^{N_{j}}=k_{j}t+B_{j}$$

Equation (2) requires two parameters to describe a sigmoid: the exponent *N_j_* describes aspects of the nonlinear behavior with respect to time, while 1/*x*_*j*,inf_ describes the value of 1/*x_j_* that could be achieved if milling were carried on for an infinite time. Away from the asymptote which emerges after prolonged milling, the expression 1/*x*_*j*,inf_ − $1/x_{j}$ at 1/*x*_*j*,inf_ is valid, and Equation (2) reduces to Equation (1) following some algebraic manipulations. In Appendix A, we also present a derivation of Equation (2) based solely on breakage kinetics without any transforms.

### 3.2. Model Development for the Apparent Breakage Rate Constant

We now develop models for the functional dependence of the apparent breakage rate constant *k_j_* on the operational–design parameters of wet bead milling and bead properties. We describe here three models with varying levels of complexity. We start by describing the development of Model A, which is a mechanistic model derived from microhydrodynamic theory (Section 3.2.1) that requires significant effort to parameterize. Following that, the development of a more computationally efficient semi-mechanistic Model B is described (Section 3.2.2). Model B is a practical, generalizable, semi-mechanistic model that can be used to describe the wet bead milling process development across mills and scales. Finally, a semi-mechanistic model, fitted to the milled particle size data covering the model compounds in this study (Model C), is presented in Section 3.2.3. Model C is a simplified variant of Model B requiring minimum number of milling experiments to parameterize the model (Section 3.2.3). This latter approach (Model C) offers a computationally and experimentally efficient modeling framework compared to fully mechanistic approaches, making it attractive for industrial simulations within timescales of interest. A high-level comparison of the modeling approaches described in this work is presented in Table 2. With these three models, we can effectively describe particle size evolution during wet bead milling for multiple drug products across mill scales.

#### 3.2.1. Model A: A Microhydrodynamics-Based Model

As small molecule organic materials are relatively brittle and easy to break compared to inorganic materials such as ores and minerals, their breakage kinetics are expected to be governed by the stressing frequency rather than the stress intensity, unless the latter is extremely low. Several in-depth studies using the microhydrodynamic (MHD) theory have concluded that the breakage kinetics during wet bead milling are governed by the average frequency of drug particle compression *a* [29,48]. Hence, our starting point in developing this model was to assume that the extent of milling *k_j_t* for a given drug nanosuspension formulation processed in a recirculation mill is given by *k_j_t* = $A_{j}^{*}$N_t_*aτ*_m_, where $A_{j}^{*}$ is a constant dimensionless parameter that is presumed to correlate with the brittleness of APIs [57,58], *N*_t_ is the number of turnovers defined earlier, and *τ*_m_ is (the single-pass) mean residence time of the suspension in the mill with volume *V*_m_ containing a true volume fraction *c* of beads in the milling chamber. Inserting the definitions of *N*_t_, *τ*_m_ = *V*_m_(1 − *c*)/*Q* into Equation (2) and noting $\gamma_{API}$ = $m_{API}/V_{tb}$, we arrived at Equation (3):

(3) $${Yt}_{j}=A_{j}^{*}a\frac{\gamma_{API}V_{m}(1-c)}{1000m_{API}}t+B_{j}$$

where $\gamma_{API}$ was the mass concentration of the drug in g/mL and 1000 was a unit conversion factor since *m*_API_ was in kg. Equation (3) shows that the apparent breakage rate constant *k_j_* equals the pre-factor in front of *t*; one may consider $A_{j}^{*}$a as the true breakage rate constant because *V*_m_(1 − *c*)*t/V*_tb_ appears to be the effective milling time in recirculation milling, also known as mean residence time of the circuit (mill and holding tank).

According to the microhydrodynamic theory [28,29,59], the average frequency of drug particle compressions between the beads *a* is calculated by multiplying the probability of a single drug particle to be caught by the beads *p* and the average oscillation frequency of a single bead ν, and is a function of bead material density *ρ_b_*, Poisson’s ratio *PR_b_*, Young’s modulus *YM*_b_, and diameter *D_b_*, as well as the granular temperature *θ*, which is dependent on both operation–design variables and drug suspension properties such as apparent shear viscosity (refer to Appendix B for the principal equations of the microhydrodynamic theory). Inserting *a* (refer to Equation (A13) in Appendix B) into Equation (3), we obtain Equation (4), or Model A, which is the most general microhydrodynamics-based model for the prediction of the particle size evolution during wet bead milling.

(4) $${Yt}_{j}=A_{j}^{**}\frac{\gamma_{API}V_{m}}{m_{API}}c^{2}{\left[1-{\left(\frac{c}{c_{lim}}\right)}^{0.33}\right]}^{-1}{\left[\frac{\rho_{b}\left(1-{PR}_{b}^{2}\right)}{{YM}_{b}}\right]}^{0.4}\frac{\theta^{0.9}}{D_{b}^{2}}+B_{j}$$

Determining the granular temperature *θ* for different operational and design conditions within the context of the microhydrodynamic theory entails significant effort and is outside the scope of this manuscript. The calculation of *θ* for a lab-scale mill for various drugs, stirrer speeds, bead loadings, bead types and sizes, etc., is described in earlier MHD studies [16,20,29,48]. However, this approach may not be practical as it requires accurate measurements of power *P* and power density *P*_v_ = *P/V*_m_ in mills at various scales, as well as density and apparent shear viscosity of the suspensions. Previous studies showed that the granular temperature *θ* varies with the rotational speed *ω* of the rotor [20,29,48,49,50,60], the volume fraction of the beads in the suspension *c* [20,29,48,49,50], the density of the beads *ρ*_b_ [29,48,49], the diameter of the beads *D*_b_ [20,50,60], and the apparent shear viscosity of the suspension *μ*_s_ at the reference temperature [16]. The density of the suspensions *ρ*_s_ is another factor that has an impact on *θ* (Appendix B). While the comparative impacts of the process variables and bead type/size on *θ* were established in the aforementioned studies, a mathematical expression to calculate *θ* directly based on process variables has not yet been formulated.

#### 3.2.2. Model B: A Practical Semi-Mechanistic Model for Wet Bead Milling

In this section, we approach the wet bead milling modeling from a semi-mechanistic standpoint describing the development of semi-mechanistic Model B. In Section 4, we will discuss fits to Model B and specific case studies which were used to support the structure of this model. Influenced by Model A and prior knowledge on parameters with potential impact on the granular temperature as discussed in Section 3.2.1, we formulated a semi-mechanistic model (Model B) with a power-law structure as described in Equation (5).

(5) $${Yt}_{j}=A_{j}\frac{\gamma_{API}V_{m}}{m_{API}}\frac{{\left(\frac{\omega }{\omega_{ref}}\right)}^{N_{1}}BL^{N_{2}}{\left(\frac{\rho_{b}}{\rho_{YSZ}}\right)}^{N_{3}}{\left(\frac{1-{PR}_{b}^{2}}{{YM}_{b}}\right)}^{0.4}D_{a}^{N_{5}}}{{\left(\frac{D_{b}}{D_{b,ref}}\right)}^{N_{4}}}\;\;{\left(\frac{\mu_{s}}{\mu_{s,ref}}\right)}^{N_{6}}{\left(\frac{\rho_{s}}{\rho_{s,ref}}\right)}^{N_{7}}{Kt}_{j}Et+B_{j}$$

Model B incorporates all the important process parameters governing milling. Similar to Model A, it is a function of the API mass *m*_API_ and concentration *γ*_API_, the mill volume *V*_m_, rotational speed *ω*, the bead loading *BL* (*BL = c*/*c*_lim_), the density of the beads *ρ*_b_, the Poisson’s ratio of the beads *PR*_b_, the Young’s Modulus of the beads *YM*_b_, the diameter of the beads *D*_b_, the apparent shear viscosity of the suspension at the reference temperature *μ*_s_, and the density of the suspensions *ρ*_s_. While the terms with parameter groups (*m*_API_, *γ*_API_, *V*_m_) and (*PR*_b_, *YM*_b_) have been transferred directly from Model A, other parameters were raised to power *N*. Since these quantities can span orders of magnitude, we normalized each of these terms by a standard reference quantity to ensure that each term remains around an order of magnitude of one. Two additional parameters (Kt and *E*) were included in Model B. We introduced Kt as a factor that accounts for temperature change from a reference temperature (15 °C) during milling, which is an Arrhenius function with a *K*2*_j_* parameter to be fitted $\exp\left[-{K2}_{j}\left(\frac{1}{273.15+\left(T\;℃\right)}-\frac{1}{288.15}\right)\right]$. To account for different milling efficiencies across different pieces of milling equipment, we also introduced a mill-scale efficiency factor, *E*, which is further discussed in Section 4.3.2. *E* accounts for the energy transfer efficiency of any mill with respect to a reference mill, which captures the impact of mill design differences at different scales; it is constant for a given mill and independent of the API formulation. Model B can be fitted to particle size data from different milling experiments with varying process conditions to obtain the corresponding unknown parameters (i.e., *A_j_*, *N*_1_, *N*_2_, *N*_3_, *N*_4_, *N*_5_, *N*_6_, *N*_7_, *K*2*_j_*, *B*_j_). The reference values for these parameters were set as 1000 rpm, 0.63 (the packing limit of the beads), 6000 kg/m^3^ (the density of the YSZ beads), 0.33 mm (a commonly used bead size in our studies), and 1.13 cP (viscosity of water at 15 °C), respectively. The unit for *A*_j_ in Model B depends on the exponent of the tip speed *N*_5_ ($kg^{0.4}\;m^{-N5-0.4}s^{-1.8}$). The connectivity between Equations (3) and (5) is explained in Appendix C.

To ensure that *θ* remains invariant upon scale-up from a reference mill, the power density *P*_v_ must remain invariant according to the microhydrodynamic theory. The asymptotic scaling analysis in Appendix B suggests a scaling of the form $\theta \propto P_{v}^{0.7}$ for fully (upper) turbulent flow. In the upper turbulent flow regime, for which Reynolds number $Re>2\times 10^{5}$, the following scaling is applicable [61]: $P_{v}=P/V_{m}\propto \omega^{3}D_{a}^{3}/L$, where *L* is the effective length of the mill chamber. This scaling led us to $\theta \propto \omega^{2.1}D_{a}^{2.1}/L^{0.7}$ and $\theta^{0.9}\propto \omega^{1.9}D_{a}^{1.9}/L^{0.6}$; therefore, as per Equation (5) to Equation (6), *N*_1_ = *N*_5_ emerged; thus, we fitted only *N_1_*–*N*_4_ independently. As will be shown below, fitting data across scales yielded $N_{1}=N_{5}≅2$, supporting the microhydrodynamic result of *N*_1_ = *N*_5_ = 1.9. For the lower turbulent flow regime ($3.5\times 10^{4}<Re<2\times 10^{5}$), using the same scaling $\theta \propto P_{v}^{0.7}$, we found *N*_1_ = 1.8 and *N*_5_ = 1.6, which are not far from the fitted values. It must be noted that in the lower turbulent flow regime, *θ* may follow *P*_v_^0.7^–*P*_v_^0.8^ scaling as some minor viscous effects emerge (for laminar flow $\theta \propto P_{v}$ scaling applies). When $\theta \propto P_{v}^{0.8}$ was used, *N*_1_ = 2.0 and *N*_5_ = 1.9 were obtained for the lower turbulent regime. The conclusion from this analysis, upon consideration of all complexities of the fluid flow in the mill and the assumptions made in the asymptotic scaling analysis, is that the microhydrodynamic model suggests $N_{1}≅N_{5}≅2$.

#### 3.2.3. Model C: Semi-Mechanistic Milling Model Fitted to Milled Particle Size Data

Equation (5) was used along with the Yt transform in Equation (2) to fit the reciprocal of *x*_10_, *x*_50_, and *x*_90_ for various model compounds in this work and estimate the model parameters, i.e., *N*_1_–*N*_4_, *A_j_*, *B_j_*, *N_j_* and *x*_*j*,inf_. The resultant equation is given in Equation (6), which we refer to as Model C, where exponents *N*_1_–*N*_4_ were determined as described in Section 4. While Model C can be applied towards future datasets without re-fitting the exponents, unlike Model B, making it the most computationally and experimentally efficient model, it bears the signatures of the specific mills and formulations used in this study. In Model C/Equation (6), *A_j_*, *B_j_*, *N_j_*, *x*_*j*,inf_, and *K*2*_j_* are the fitting parameters and vary with the size quantiles, API, and formulation. Parameters were obtained through nonlinear fitting.

(6) $$Yt_{j}={\left(\frac{1/x_{j}}{1/x_{j,inf}-1/x_{j}}\right)}^{N_{j}}=A_{j}\frac{\gamma_{API}V_{m}}{m_{API}}\frac{{\left(\frac{\omega }{1000}\right)}^{2}BL^{3}{\left(\frac{\rho_{b}}{6000}\right)}^{1.4}{\left(\frac{1-{PR}_{b}^{2}}{{YM}_{b}}\right)}^{0.4}D_{a}^{2}}{{\left(\frac{D_{b}}{0.33}\right)}^{0.3}\;}{Kt}_{j}Et+B_{j}$$

If multiple mill scales were used during the Design of Experiments (DoE) and the efficiencies of the mills are unknown, Equation (6) can be used with a match function for the mill-scale efficiency factor *E* term, which can be fitted to the experimental data to estimate efficiencies of different mills. This procedure will be discussed in Section 4.3. The efficiencies for DV50, DV150, DV300, DV2000 and DV4000 mills were estimated as 0.70, 1.00, 0.83, 0.24 and 0.19, respectively. If readers are interested in estimating the efficiencies for mills that are different from the specific Netzsch mills used in this study (see Table 1) but have similar chamber and impeller geometry, they can apply the empirical relationship shown in Equation (7) to estimate *E* (refer to Section 4.3 for more details). Here, log E in Equation (7) is a function of the agitator diameter *D*_a_ and length *L*_a_.

(7) $$\log E=-14-5.1\log D_{a}+2.0\log\left(\frac{L_{a}}{D_{a}}\right)+8.1\left[\left(\log D_{a}+2.4\right)\times \left(\log\left(\frac{L_{a}}{D_{a}}\right)-0.111\right)\right]$$

In addition, if milling was not run for too long to approach the apparent grinding limit (i.e., a significant fit for *x*_j,inf_ cannot be obtained), readers can estimate *x*_j,inf_ via an empirical relation (Equation (8)) with coefficient *C*_j_ as 0.057, 0.088 and 0.125 for *x*_10_, *x*_50_ and *x*_90_, respectively (refer to Section 4.3 for more details).

(8) $$x_{j,inf}=C_{j}{BL}^{-0.25}D_{b}^{0.12}{YM}_{API}^{0.24}$$

With the additional flexibility provided by Equations (7) and (8), Model C (Equation (6)) is a convenient model that can be parameterized with as few as one experiment for a specific drug product and formulation. It can then predict the particle size and milling time required to reach a target particle size for any process condition across milling scales (from lab to pilot or commercial scales).

### 3.3. Reversing the Transform

When Equation (1) was used as the linearizing transform, reversing the transform was straightforward: the *Yt* function was raised to the power of (1/*N_j_*) to then calculate 1/*x_j_* at different time points. When Equation (2) was used as the linearizing transform, Equation (9) was used to calculate the reciprocal particle size:

(9) $$\frac{1}{x_{j}}=\frac{{Yt}_{j}^{1/N_{j}}\frac{1}{x_{j,inf}}}{1+{Yt}_{j}^{1/N_{j}}}$$

Using statistical analysis software with nonlinear solving capabilities like JMP version 17, it is possible to substitute Equation (4), Equation (5), or Equation (6) for the *Yt* term in Equation (9) and directly solve for the reciprocal particle size. In this study, Model B (Equation (5)) was fitted in JMP in this way with reasonable guesses for each parameter as starting points. The reverse-transformed Equation (5), with reference values substituted following Equation (9), would be as shown in Equation (10).

(10) $$\frac{1}{x_{j}}=\frac{{\left[A_{j}\frac{\gamma_{API}V_{m}}{m_{API}}\frac{{\left(\frac{\omega }{1000}\right)}^{N_{1}}BL^{N_{2}}{\left(\frac{\rho_{b}}{6000}\right)}^{N_{3}}{\left(\frac{1-{PR}_{b}^{2}}{{YM}_{b}}\right)}^{0.4}D_{a}^{N_{1}}}{{\left(\frac{D_{b}}{0.33}\right)}^{N_{4}}}\;\;{Kt}_{j}Et+B_{j}\right]}^{1/N_{j}}\frac{1}{x_{j,inf}}}{1+{\left[A_{j}\frac{\gamma_{API}V_{m}}{m_{API}}\frac{{\left(\frac{\omega }{1000}\right)}^{N_{1}}BL^{N_{2}}{\left(\frac{\rho_{b}}{6000}\right)}^{N_{3}}{\left(\frac{1-{PR}_{b}^{2}}{{YM}_{b}}\right)}^{0.4}D_{a}^{N_{1}}}{{\left(\frac{D_{b}}{0.33}\right)}^{N_{4}}}\;\;{Kt}_{j}Et+B_{j}\right]}^{1/N_{j}}}$$

The term Kt*_j_* in the above equation was derived as an Arrhenius equation in Section 3.2.2, but in the interest of brevity, this substitution is not shown explicitly. In our studies, we fitted Equation (10) directly to the data from our case studies, solving for the product-specific terms *A_j_*, *B_j_*, *N_j_*, *x*_*j*,inf_ and the temperature term *K*2*_j_* for each size quantile and the nonlinear exponents (*N*_1_ − *N*_4_) on *ω*, *BL*, *D*_b_, and *ρ*_b_, which were shown to be consistent in value across all studies and thus product independent, as will be illustrated in Section 4.1 and Section 4.2. We note that not all power-law exponents can be estimated in every case study, as the relevant process parameter might not have been investigated in the experimental design, in which case the associated exponents were fixed to those in Equation (6) (Model C). A model of this complexity required several case studies to establish validity across numerous products and five different scales as described Section 4.1 and Section 4.2.

## 4. Results and Discussion

In Section 4.1, an overall summary of the Model B (Equation (5)) fits and the impact of process parameters on the particle breakage will be discussed. In support of Section 4.1, Section 4.2 will illustrate the specific examples from case studies involving different drug products. Finally, Section 4.3 will show how Model C was used to derive mill efficiency correlations based on mill properties (Equation (7)); grinding limit correlations based on process and material properties (Equation (8)); and milling rate correlations based on material properties.

### 4.1. Impact of Process Parameters on Particle Size: Journey from Model B to Model C

The exponents of Model B were found by fitting Equation (5) to particle size data from a number of wet bead milling studies, but since not all studies had variations on all *w*, *BL*, *D*_b_, and *ρ*_b_ process parameters, Model C was obtained using insights from all case studies collectively. Some of the process parameters, such as rotor speed and bead loading, were investigated in multiple case studies. The exponents obtained for the different particle size quantiles (*x*_10_, *x*_50_, and *x*_90_) have some variation. In each of the subsequent subsections, we summarize the fitted values of the *N*_1_, *N*_2_, *N*_3_ and *N*_4_ exponents. When parameterizing Model C, we only recorded the fitted values of the exponents if all parameter estimates were statistically significant in the model. If the coefficient estimates were more than two standard deviations away from zero, the estimates were identified as statistically significant.

#### 4.1.1. Impact of Rotor Speed

The rotational rotor speed *ω* (in rpm) is one of the most important and impactful process parameters [32,41] for wet bead milling. It increases the breakage rate, as it provides more frequent and stressful collisions [48]. Our microhydrodynamic analysis suggests that ω should have an exponent of 2. We tested this hypothesis in the case studies described in Section 4.2.1, Section 4.2.2 and Section 4.2.3 with Model B (Equation (5)). A summary of the findings for all studies is presented in Table 3, which shows *N*_1_ ≅ 2 is the most frequent exponent, which agrees well with an earlier scale-up study in Ref. [47]. The value of 1.39 found for DP1 could be due to a tendency to aggregate at certain mill scales, which takes longer than expected to break down.

#### 4.1.2. Impact of Bead Loading

Bead loading *BL* has been shown to be the most impactful parameter for particle breakage in the literature [29,47,48]. An increase in *BL* increases the number concentration of the beads and the value of the radial distribution function at contact, decreases the inter-bead distance, and dramatically increases the number of bead–bead collisions, which ultimately increases the frequency of drug particle compressions and the apparent breakage rate constant [20,29]. The studies described in Section 4.2.1, Section 4.2.2 (Figure S1), Section 4.2.3 (Figure S2) and Section 4.2.4 are summarized in Table 4 with the bead loading range explored in each study, the fitted exponent for bead loading with Model B (Equation (5)), and the relative milling rate due to bead loading ($BL^{N_{2}}$), which accounts for the differences in the milling rate resulting from the different bead loading levels. Since there were only two levels of bead loading in the DP3 dataset, 85% and 99.8%, and a different exponent was observed between the two of only 1.29, the relative milling rate at 99.8% was calculated as a ratio to the rate at 85% using the following equation: ${85\%}^{2.89}\frac{{99.8\%}^{1.29}}{{85\%}^{1.29}}$, where 1.29 is the exponent obtained in the DP3 study, and 2.89 was obtained in the DP1 study which used 85% bead loading. Only parameter fits that were significant were included in this analysis.

**Figure 2:**
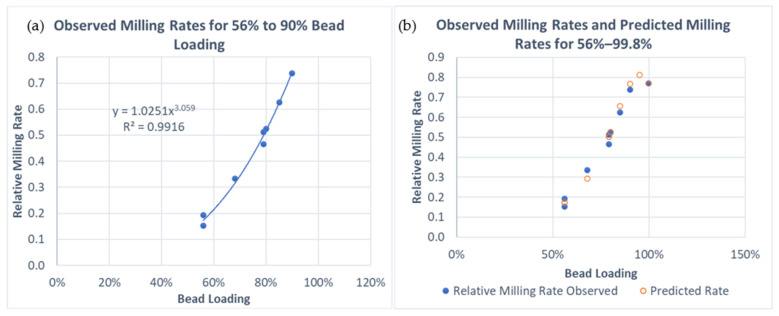
(**a**) Observed milling rates for 56–90% bead loading following a cubic relationship. (**b**) Observed and predicted milling rates for 56–99.8% bead loading following a more complex relationship (Equation (11)).

When the relative milling rate due to bead loading is plotted versus bead loading (Figure 2), a nearly perfect cubic relationship is observed over all studies carried out using 56–90% bead loading. The DP1 and DP3 studies at DV300 scale added the complexity that the bead loading was 99.8%, near the maximum packing limit in the mill, where some level of inefficiency was observed. In addition, 90% bead loading and 99.8% bead loading did not lead to markedly different observed milling rates.

If the process stays within 56–90% bead loading, then the simple cubic term on bead loading is appropriate (i.e., *N*_2_ = 3), but in excess of 90% bead loading, a more complex term instead of *BL^N^*^2^ is needed, as shown in Equation (11).

(11) $$BL^{N_{2}}=\frac{1}{1.23+30.0{\left(BL-95.0\%\right)}^{2}}$$

where “*BL*” is the bead loading expressed as a percentage. Guner et al. [29] showed that the impact of bead loading becomes more significant when bead loading approaches the packing limit since beads become much closer to each other, as shown by the radial distribution function. However, bead loading beyond 95% was not explored in that study, and the mills used in this study seem to have an efficiency loss when bead loading was higher than 95.0%, as implied by Equation (11). Hence, the right-hand side of Equation (11) was substituted in place of *BL*^3^ in Equation (6) for studies where *BL* exceeded 90%.

#### 4.1.3. Impact of Bead Material

Density is an important property of the bead materials as it directly affects the energy input to the process, and therefore collision frequency and stress [29,48]. A detailed microhydrodynamic analysis suggests that because these have higher density, Yttrium-stabilized zirconia (YSZ) beads induce more frequent and forceful collisions than crosslinked polystyrene (CPS) beads, which favors drug particle breakage as signified by the higher apparent breakage rate constant when using YSZ beads [29,48]. Bead density in our model was considered a ratio to the density of the YSZ beads (6000 kg/m^3^), as it is the most preferred bead material [25]. Normalized bead density was raised to the 1.4 power, ${\left(\frac{\rho_{b}}{\rho_{YSZ}}\right)}^{1.4}$, as per results from a study completed at NJIT comparing milling rates of crosslinked polystyrene (CPS) beads to YSZ beads [29,48,49]. CPS beads in this study were noted to have a density of 1040 kg/m^3^. This study is discussed in more detail in Section 4.2.2. The power of 1.4 was obtained by averaging the *N*_3_ fits to *x*_50_ and *x*_90_ size classes with Model B (Equation (5)), as the *x*_10_ fit was insignificant due to noise. The Young’s modulus *YM*_b_ and the Poisson’s ratio *PR*_b_ of the beads are 200 GPa and 0.20 for the YSZ beads [62] and 1.5 GPa and 0.33 for the CPS beads [63].

#### 4.1.4. Impact of Bead Size

Bead size can be an important process parameter as it may provide combined advantages of faster breakage [21,50,64], lower heat generation rate [65,66,67], and lower media wear [21,64]. However, its impact on the breakage rate may be more subtle because it has counteracting microhydrodynamic trends where smaller beads capture drug particles more frequently but apply less stress upon contact [20,60]. Therefore, the exponent for the bead size term was small, at only 0.3 in our model. The normalized bead size with the reference 0.33 mm bead size, corresponding to a nominal size of 300 µm, affects the apparent breakage rate constant *k_j_* through ${\left(\frac{D_{b}}{0.33}\right)}^{0.3}$. The impact of bead size was rather weak compared to other parameters; so, a carefully designed DoE and data with minimal noise are required to detect this relationship with a significant model fit. Among the studies in this paper, only an NJIT study in Section 4.2.3 and a DP3 study in Section 4.2.4 varied the bead sizes. However, since the DP3 data did not have enough degrees of freedom, and the NJIT study had noise in the *x*_10_ and *x*_90_ data, only the *x*_50_ fit with Model B (Equation (5)) provided an accurate prediction for the bead size exponent. Even though a 0.3 fit was obtained by only one quantile particle size of one drug, the fixed 0.3 exponent worked well for both drugs and all quantiles.

#### 4.1.5. Impact of Temperature

Temperature during milling varies over time and is influenced by the process parameters [65,66,67], which may have an impact on the particle size evolution. To investigate this hypothesis, we proposed an Arrhenius function for the *j*^th^ size quantile that accounts for the temperature dependency, i.e., Kt*_j_* (see Section 3.2.2). *K*2*_j_* is a fitted Arrhenius parameter that accounts for the temperature effect for the *j*^th^ quantile. At the reference temperature of 15 °C, Kt*_j_* will normalize to a value of one. This prevents uncertainties in the estimate of *K*2*_j_* from affecting the error estimate of *A_j_*. The Arrhenius parameter *K*2_j_ is analogous to an activation energy divided by the universal gas constant *R* and is likely affected by several competing temperature-dependent processes that are all exponential with respect to temperature, including changes in viscosity, Young’s modulus, and possibly particle growth mechanisms like Ostwald ripening, where higher temperatures and temperature swings can dissolve the finer particles and recrystallize the material on the larger particles. In general, milling should speed up with a reduction in formulation viscosity that would tend to occur with increasing temperature; however, this is often balanced or overshadowed by the faster aggregation–Ostwald ripening rates at higher temperatures [68], or slower breakage rates associated with changes in Young’s modulus [69]. Temperature does vary somewhat within each run. For this reason, the time-averaged temperature at the *i*^th^ timepoint (*T*avg*_i_*) was used with Model B and Model C fits (Equation (12)).

(12) $$Tavg_{i}=\left[Tavg_{i-1}*t_{i-1}+\left(\frac{T_{in}+T_{out}}{2}\right)*\left(t_{i}-t_{i-1}\right)\right]/t_{i}$$

where *t*_i_ is time at the *i*^th^ time point, and *T*_in_ and *T*_out_ are the inlet and outlet temperatures to the mill. This equation was used to calculate the time-averaged value up to each point in time for model calculation. In general, GSK sees a trend that milling rates speed up at cooler temperatures [70]; so, negative values of *K*2*_j_* are estimated during model fitting. It is possible that systems with higher viscosity may exhibit a dominant temperature-dependent viscosity effect, and milling becomes faster at increased temperatures, with resulting fits for *K*2*_j_* being positive. Future studies examining more formulation effects than those considered in this paper might require breaking Kt*_j_* up into separate viscosity, Young’s Modulus, and particle growth effects. If the formulation does not change, then Kt*_j_* alone appears to be a sufficient descriptor for the temperature effect. We demonstrated successful *K*2*_j_* estimations for DP1 and DP5, as shown in Section 4.2.1 and Figure S15, respectively.

### 4.2. Case Studies in Support of Model C Development

#### 4.2.1. Impact of Rotor Speed, Bead Loading, Temperature at DV2000 Scale (DP1)

The first version of Model B was developed at GSK in support of Drug Product 1 (DP1). After the development of the final version of Model B (Equation (5)), we first fitted it to a dataset coming from a Design of Experiments (DoE) exploring the impacts of tip speed and bead loading, as well as supplemental runs where batch size was changed from 4 kg to 2 kg API mass. This eight-run set of experiments was completed using the DV2000 wet bead mill where the tip speed was varied from 5 to 6 m/s, and bead loading was varied from 80% to 90%. The average temperature among runs also varied from 11.8 to 19.6 °C. Pumping rate *Q* was set based on *V*_m_ at one mill volume per minute flowrate. The product team sampled several of the batches with a relatively large sample size, taking 18% of the batch mass in a sample point an hour before completion of milling. For this reason, the time-averaged batch mass (*Average_Mass_i_*) was used with Model B and Model C fits for this drug product (Equation (13)), similar to what we did with the time-averaged temperature.

(13) $${Average\text{\_}Mass}_{i}=\left[Average\text{\_}Mass_{i-1}*t_{i-1}+\left({Mass}_{i}\right)*{(t}_{i}-t_{i-1})\right]/t_{i}$$

We have seen in this dataset that an increase in tip speed (or rotation speed for a given mill) and bead loading both led to higher *T*avg. This finding is in line with earlier heat transfer–generation studies [65,66,67], which demonstrated that a higher power consumption was observed at the higher rotation speed, and that higher bead loading was the origin of higher heat generation and ensuing temperature rise.

**Figure 3:**
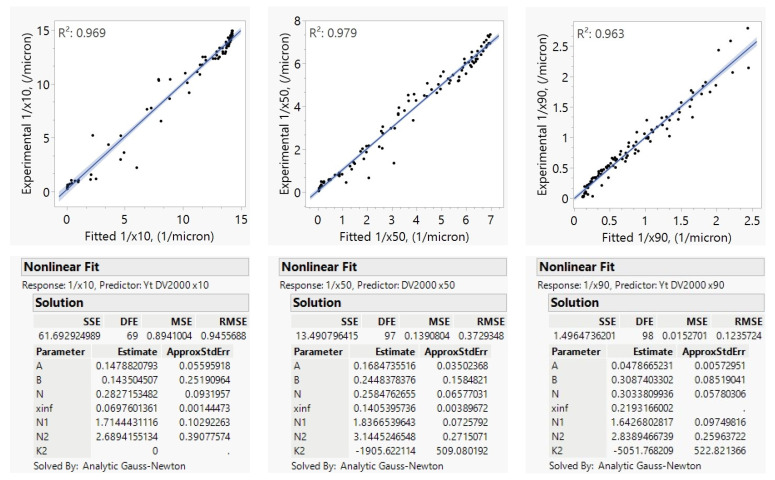
Model fits and parity plots for DP1/DV2000 dataset for 1/*x*_10_, 1/*x*_50_, 1/*x*_90_ from Model B (Equation (5)), with *N*_3_ = 1.4, *N*_4_ = 0.3, and *N*_5_ = *N*_1_.

YTZ beads of 300 µm nominal size were used throughout the experiments; thus, exponents of the bead density and bead size terms were not explored in this case study. Since only one mill scale had been used here, the mill-scale efficiency factor (*E*) was not explored either. The exponents of the parameters that were not explored in this case study were set to those from Model C, which was developed based on a combination of case studies as summarized in Section 4.1. Model B, as shown in Equation (5) with *N*_3_ = 1.4 and *N*_4_ = 0.3 and *N*_5_ = *N*_1_, was fitted to the reciprocal particle size quantiles to find the ω and BL exponents, *N*_1_ and *N*_2_. Milling was not carried out for long enough to find an *x*_*j*,inf_ fit for the *x*_90_ population; so, this was estimated as 0.219 µm using Equation (8) for the DV2000 dataset. The resulting model parameter fits and model goodness-of-fit (*R*^2^ values) as calculated by JMP version 17 using analytic Gauss Newton nonlinear solver for the prediction of 1/*x*_10_, 1/*x*_50_, and 1/*x*_90_ for the DV2000 dataset are shown in Figure 3. In the JMP figures, the dots are for the experimental data, the lines are for the model fit and shades are for the error of the fit.

The deviations in the parity plots (Figure 3) appear random, which indicates that the $\frac{\gamma_{API}V_{m}}{m_{API}}$ term could capture the impact of the change in API mass well. The fits for the exponent *N*_1_ for the prediction of 1/*x*_10_, 1/*x*_50_, and 1/*x*_90_ consistently showed values in the range of 1.6 to 1.9. The bead loading *BL* was noted to have an exponent *N*_2_ in the 2.6–3.2 range. The *N*_1_ and *N*_2_ fits slightly varied among different case studies, as discussed in Section 4.1, since those exponent fits were influenced by measurement and sampling errors, and the fitted numbers in this case study have averages of approximately *N*_1_ = 2 and *N*_2_ = 3 as in Model C. Most interestingly, the negative *K*2_j_ values suggest that milling proceeded significantly faster at lower temperatures, and this effect was more pronounced for the coarser sizes (*x*_90_ vs. *x*_50_), with no temperature impact for *x*_10_. This drug product is known to exhibit Ostwald ripening, which proceeds more quickly at warmer temperatures [68]. This mechanism may explain why *x*_90_ is more strongly impacted by temperature than *x*_10_ and *x*_50_. The overall goodness-of-fit was 96.9%, 97.9%, and 96.3% for the 1/*x*_10_, 1/*x*_50_, and 1/*x*_90_, respectively.

This drug product was scaled up from non-DoE experiments at DV300 scale, to DoE experiments at DV2000, and to multiple runs at DV4000 scale where batch size varied between 10 kg and 30 kg of API, and tip speed varied from 5.5 to 6.5 m/s. Flow rates through the mill were also changed from 2 L/min to 4 L/min at each batch size change. This resulted in 26 runs across three scales. Since the DV300 data included batches with 99.8% bead loading, the more complex version of the bead loading term shown in Equation (11) was used instead of *BL*^3^. In some of the DV4000 experiments, the suspension was diluted, causing a change in the drug loading. In this case, we used time-averaged drug loading (${\gamma_{API,avg}}_{i}$) in Equation (14), where ${\gamma_{API}}_{i}$ is the current API loading in the system, similar to what we did with the time-averaged temperature.

(14) $${\gamma_{API,avg}}_{i}=\left[{\gamma_{API,avg}}_{i-1}*t_{i-1}+\left({\gamma_{API}}_{i}\right)*{(t}_{i}-t_{i-1})\right]/t_{i}$$

**Figure 4:**
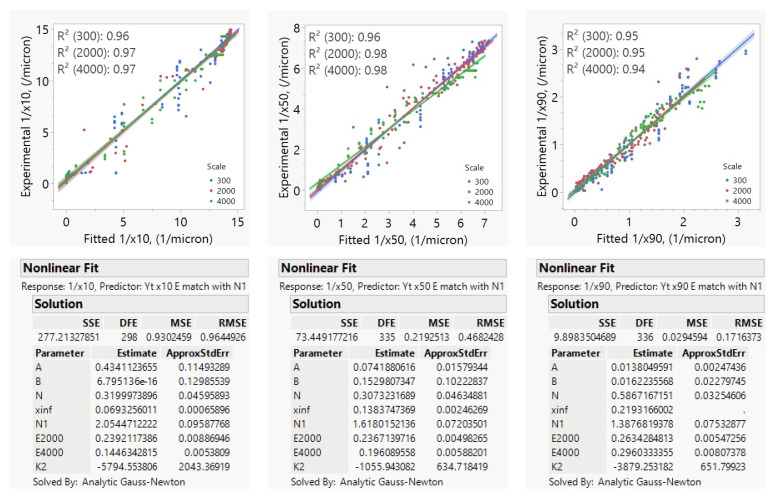
Model fits and parity plots for DP1 at three scales—DV300 (blue), DV2000 (red), DV4000 (green)—for 1/*x*_10_, 1/*x*_50_, 1/*x*_90_ from Model B (Equation (5)) with bead loading term as in Equation (11), instead of BL*^N^*^2^, *N*_3_ = 1.4, *N*_4_ = 0.3, and *N*_5_ = *N*_1_.

Model B (Equation (5)) was used to model this set of experiments, with *N*_3_ = 1.4, *N*_4_ = 0.3 and *N*_5_ = *N*_1_ and fitted mill efficiencies. The “match” function in JMP was used to assign parameters to each mill scale, and the E2000 and E4000 terms were estimates of the mill-scale efficiency factor *E* at the DV2000 and DV4000 mill scales. Here, the DV300 was assigned an efficiency value of 0.83, which is the efficiency estimate of DV300 as described in Section 4.3, and the E2000 and E4000 parameters then describe the relative efficiencies for the DV2000 and DV4000 mL mills as compared to the DV300. The resulting parameter fits for the 1/*x*_10_, 1/*x*_50_, 1/*x*_90_ models and parity plots at each scale are shown in Figure 4.

We found that the model fit well across every scale considered in the DP1 study with *R*^2^ values ranging from 0.95 to 0.98. Mill-scale efficiency factors did vary by the particle size quantile considered with each mill scale. DV2000 and DV4000 mills were found to be less efficient since their tip diameters are larger than the small-scale mills. When scaling across mills, we also found that the exponent of ω (*N*_1_) may need to be fixed at a value of 2, as ω changed with scale, and some covariance can occur between the mill-scale efficiency factor estimate and *N*_1_. For this reason, the *N*_1_ was fixed at 2 and the mill-scale efficiency factors were re-estimated as shown in Figure S3 of the Supplementary Materials. This way, we obtained a better estimate of the mill-scale efficiency factors so that we could use them in Model C and Equation (11) with higher confidence.

#### 4.2.2. Impact of Bead Loading, Bead Material, Rotor Speed (NJIT Study)

This dataset followed a two-level full-factorial design of experiments (DoE) with stirrer speed (3000, 4000 rpm), bead loading (56%, 79%), and bead material (YSZ, CPS) variations. The formulation of 10% FNB, 7.5% HPC-L, and 0.05% SDS was milled with Microcer 80 mL in all runs. The details of the solid-state characterization of the milled suspension, the particle size evolution in each run, and the stability of the formulation can be found in Refs. [29,48,49]. YSZ beads have been the most preferred beads in the wet stirred media milling literature, followed by CPS beads. While YSZ beads provide more stressful and frequent collisions due to their higher density ($\rho_{YSZ}$ = 6000 kg/m^3^ vs. $\rho_{CPS}$ = 1040 kg/m^3^), CPS beads can capture more drug particles in-between as they are softer and form a larger contact circle than YSZ beads (*YM*_b_ for zirconia = 200 GPa [62] vs. *YM*_b_ for polystyrene = 1.5 GPa [63]). Even though both bead materials have the potential for providing fast breakage kinetics through different mechanisms, the collision stress and frequency outweigh the contact circle diameter, and zirconia beads provide faster breakage overall [29,48]. On the other hand, according to their lower collision stress, CPS beads can provide a gentler particle breakage, which, in turn, may reduce the amorphous transformation rates for sensitive materials [71] and contamination from media wear. In addition, they provided better control over temperature increase during milling [49], making CPS a competitive alternative to YSZ. In order to address the differences in milling rates via different bead materials, since zirconia is the most used material and density is the most important feature of the materials in terms of particle breakage rate, the relative bead density term *ρ*_b_/*ρ*_YSZ_ was used in the model. Model B (Equation (5)) with *N*_4_ = 0.3 and *N*_5_ = 2 was fitted to the experimental reciprocal particle size data.

**Figure 5:**
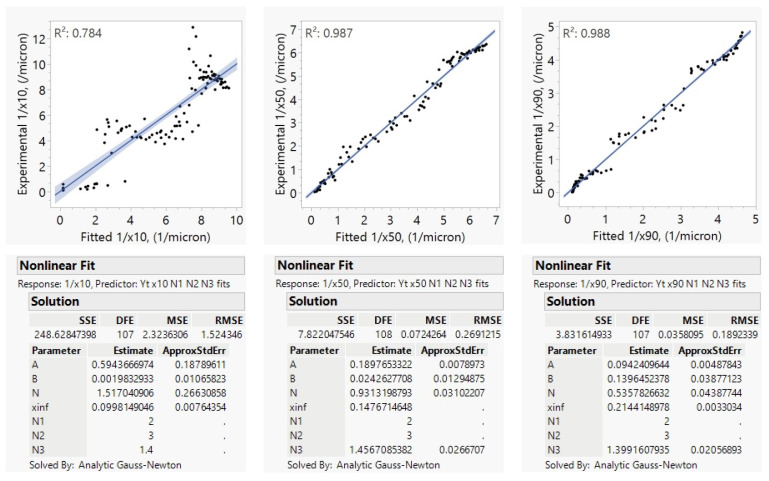
Model fits and parity plots for fenofibrate/Microcer dataset for 1/*x*_10_, 1/*x*_50_, 1/*x*_90_ from Model B (Equation (5)), with *N*_4_ = 0.3 and *N*_5_ = 2.

Figure S1 shows the fitted parameters and fitting statistics, where *x*_50_ and *x*_90_ fits were statistically significant, and the exponents of ω and *BL* (*N*_1_ and *N*_2_) for *x*_50_ were found to be 2.13 and 3.37, respectively, which compares well to all the exponents from other studies, which led to the use of 2 and 3 in Model C. The scatter in 1/*x*_10_ resulted in an insignificant model. Therefore, only the 1/*x*_50_ and 1/*x*_90_ fits were kept as the bases for *N*_1_, *N*_2_, and *N*_3_ exploration in Figure 5.

For model consistency across different case studies, *N*_1_ and *N*_2_ were fixed at 2 and 3, and the model was recalibrated for *x*_50_ and *x*_90_ as shown in Figure 5, yielding *N*_3_ of 1.46 and 1.40, respectively. Therefore, an average value of *N*_3_ = 1.4 with two significant figures was used in Model C (Equation (6)) as the power for the bead density (*ρ*_b_/*ρ*_YSZ_) term. The final equation using these fixed parameters for *N*_1_, *N*_2_, and *N*_3_ was fitted to *x*_10_ data, and the model became significant despite the noise in the data, as can be seen in the left side of Figure 5. The bead density term *ρ*_b_/*ρ*_YSZ_ = 1 when YSZ beads are used, and *ρ*_b_/*ρ*_YSZ_ = 0.17 when CPS beads are used. Hence, with *N*_3_ = 1.4, the model would predict a slower decrease in particle size during milling for polystyrene beads compared to zirconia beads under otherwise identical conditions, which is in agreement with other findings in the literature [72].

#### 4.2.3. Impact of Bead Size, Bead Loading, and Rotor Speed (NJIT Study)

**Figure 6:**
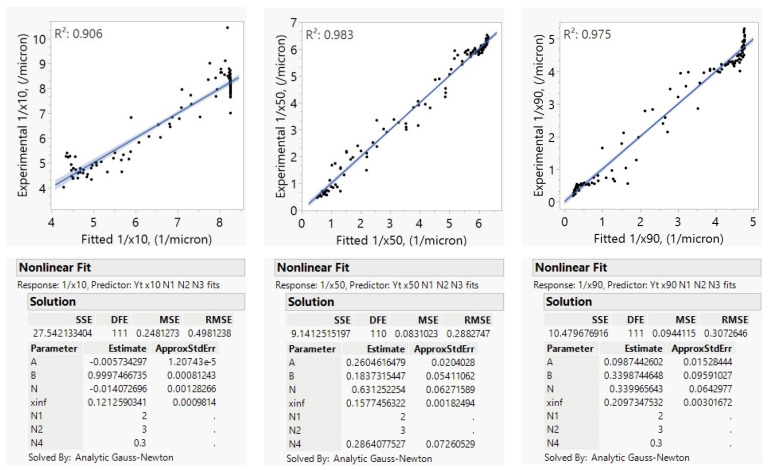
Parity plots and model fits for griseofulvin/Microcer dataset (NJIT) for 1/*x*_10_, 1/*x*_50_, 1/*x*_90_ from Model B (Equation (5)) with *N*_3_ = 1.4 and *N*_5_ = 2.

This experimental study was designed to have three different rotor speeds ω (3000, 3500, and 4000 rpm), three different bead loadings *BL* (56%, 68%, and 79%) and two different YSZ bead sizes *D*_b_ (0.2 and 0.4 mm) with a total of 10 runs. Milling was carried out for 3 h using the same mill (Microcer 80 mL located at NJIT) and the same drug formulation (10% GF, 7.5% HPC-L, and 0.05% SDS). Model B (Equation (5)) with *N*_3_ = 1.4 and *N*_5_ = 2 was used to fit the reciprocal particle sizes, allowing for the estimation of *N*_1_, *N*_2_ and *N*_4_. The impact of process parameters was assessed by fitting 1/*x_j_* data; however, since the scatter in 1/*x*_10_ and 1/*x*_90_ data was usually higher compared to 1/*x*_50_ data, the significance of the model fits was not as strong (see Figure S2 and Figure 6), similar to 1/*x*_10_ in Section 4.2.2. This could be attributed to the low level of aggregation affecting the tails of the PSD at the very high HPC-L loading. This high concentration of HPC-L was selected to increase power consumption *P* so that accurate *P* values can be used in the microhydrodynamic model in a previous study [49]. The 1/*x*_10_ model fails to have a significant rate term (*A*), whereas the 1/*x*_90_ model fails to have a significant bead size exponent term (*N*_4_), as can be seen in Figure S2 in the Supplementary Materials. Therefore, we focused on the 1/*x*_50_ model for the investigation of *w*, *BL*, and *D*_b_ impacts. The positive exponents indicate that the milling rate increased at higher ω and *BL* with the use of smaller beads. The exponents of ω and *BL* were observed to be 1.99 and 2.84, respectively, which were again very close to the exponents obtained in other studies; so, this informed the use of exponents of 2 and 3 in Model C. As can be seen in Figure 6, when the *N*_1_ and *N*_2_ values were set to 2 and 3 for *x*_50_, *N*_4_ was found to be 0.3, which is the justification of the ${\left(\frac{D_{bead}}{0.33\;mm}\right)}^{0.3}$ term in the Model C. The relative impact of process parameters, i.e., *BL* > ω > *D*_B_, on the milling rate agreed with previous studies [20,60].

Although the parameter ranges used in this dataset and those in Section 4.2.2 were the same, the drugs were different; regardless, the impact of stirrer speed ω and bead loading *BL* on the milling rate was found to be consistent.

#### 4.2.4. Impact of Rotor Speed, Bead Size, and Scale from DV150-DV300 (DP3 Study)

**Figure 7:**
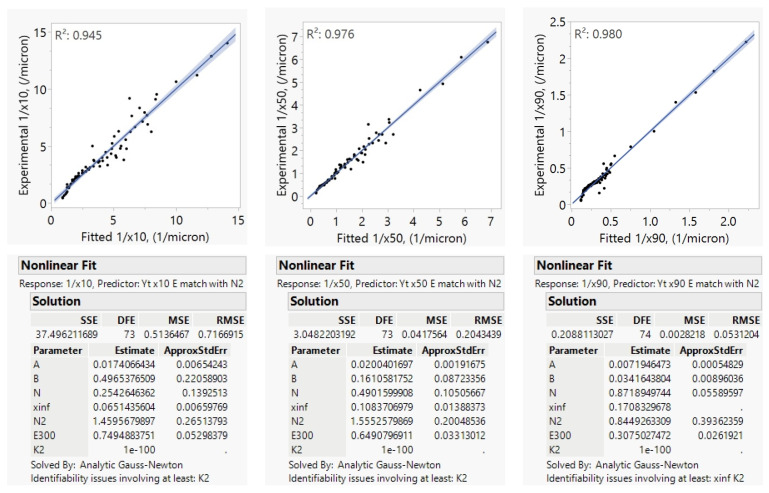
Model fits and parity plots for DP3 datasets at DV150 and DV300 scales for 1/*x*_10_, 1/*x*_50_, 1/*x*_90_ from Model B (Equation (5)), with *N*_1_ = 2, *N*_3_ = 1.4, *N*_4_ = 0.3 and *N*_5_ = 2.

In this dataset, there were six experiments in total, five of which were performed using the DV300, and one was performed using the DV150. While the DV150 experiment was carried out with 85% bead loading, 0.3 mm nominal sized YSZ beads, and 5.5 m/s tip speed, the DV300 experiments were performed with two different bead loadings, which were higher compared to previous case studies (85% and 99.8%), and with two different bead sizes (0.3, 0.65 mm), where all smaller bead studies were loaded at 99.8% and larger beads were loaded at 85%. Since the experimental design was not well suited for capturing the impact of both bead size and bead loading at the same time, we used the previously developed ${\left(\frac{D_{bead}}{0.33mm}\right)}^{0.3}$ term and fitted the bead loading exponent to understand the impact of increasing bead loading in the high bead loading range close to packing. A version of Equation (5), where *N*_1_ = 2, *N*_3_ = 1.4, *N*_4_ = 0.3 and *N*_5_ = *N*_1_, with the predicted *x*_*j*,inf_ values shown in Table 5, was fitted to 1/*x*_10_, 1/*x*_50_, and 1/*x*_90_ data, and the fit statistics together with the parity plots are shown in Figure 7. The fitted *N*_2_ values were different from previously observed values due to a possible efficiency loss at high bead loading close to the packing limit. The average of the fitted *N*_2_ values was found to be 1.29, which was used in the *BL*^N2^ analysis described in Section 4.2.1.

### 4.3. Benefits of Model C: Underlying Trends in the Factors Governing Milling Other Than Process Parameters

While fitting Model B would result in better fitting statistics since it has more degrees of freedom, a consistent model fit to a group of studies, where different APIs, formulations, and mills were used, would better elucidate whether there is any underlying behavior governing the impact of drug, suspension, and mill properties, and process conditions on apparent grinding limit, *x*_*j*,inf_, mill-scale efficiency factor, *E*, and milling rate, *A*_j_. In this section, Model C (Equation (6)) was fitted to multiple datasets so that the fitted parameters on *x*_*j*,inf_, *E*, and *A*_j_. could be compared across studies. Then, driving forces for the difference in *x*_*j*,inf_, *E*, and *A*_j_ fits were explored considering drug, process, and mill properties.

#### 4.3.1. The Apparent Grinding Limit

If the particle size does not approach the apparent grinding limit, *x*_*j*,inf_, within the given milling time, the nonlinear fitting routine will yield a lack of parameter fit for *x*_*j*,inf_ and potentially lack of significance in the *N_j_* and *A*_j_ fits, as these parameters are all somewhat covariant. Equation (1) can always be used as the linearizing transform in these cases, but it would be convenient to have a model to predict *x*_*j*,inf_ in these cases, thus allowing for the estimation of the entire sigmoidal curve. This then engables the use of the transform in Equation (2). To estimate *x*_*j*,inf_, we sought a mathematical relation for the fitted *x*_*j*,inf_ values as a function of relevant process parameters and Young’s modulus of the APIs (*YM*_API_) and arrived at Equation (8), which was previously discussed in Section 3.2.

To derive Equation (8), we first needed to obtain *x*_j,inf_ by fitting Model C (Equation (6)) to datasets for four drug products where milling was run for long enough for the particle size to approach the apparent grinding limit. We used data from multiple drug products to derive our empirical *x*_j,inf_ model to ensure that it can generalize across drug products. Since the particle size approached *x*_*j*,inf_ in all runs in the griseofulvin case study from Section 4.2.3, Model C (Equation (6)) was fitted to experimental 1/*x*_50_ values for each individual run, yielding fitted *x*_50,inf_ values. For griseofulvin, since the *x*_10_ and *x*_90_ measurements were noisy compared to *x*_50_ data, instead of having individual run fits, *x*_10,inf_ and *x*_90,inf_ were found only by fitting Model C to the complete dataset. The other three drug products where we could obtain a significant *x*_*j*,inf_ fit to the data were DP1 in Section 4.2.1, fenofibrate in Section 4.2.2 and DP3 in Section 4.2.4. The datasets are summarized in Table 5 with tip speeds *U*_tip_, bead loadings *BL*, bead sizes *D*_b_, *YM*_API_, and fitted *x*_*j*,inf_ values.

**Table 5:** Grinding limit *x*_10,inf_, *x*_50,inf_, and *x*_90,inf_ fits.

Drug	Tip Speed (m/s)	Bead Loading (%)	Bead Size (mm)	Young’s Modulus (GPa)	*x*_10,inf_ (µm)	*x*_50,inf_ (µm)	*x*_90,inf_ (µm)
Griseofulvin ^a^	11	56	0.2	11.5 [73]	N/A	0.160	N/A
Griseofulvin ^a^	11	56	0.4	11.5	N/A	0.162	N/A
Griseofulvin ^a^	11	79	0.2	11.5	N/A	0.136	N/A
Griseofulvin ^a^	11	79	0.4	11.5	N/A	0.160	N/A
Griseofulvin ^a^	14.7	56	0.2	11.5	N/A	0.159	N/A
Griseofulvin ^a^	14.7	56	0.4	11.5	N/A	0.156	N/A
Griseofulvin ^a^	14.7	79	0.2	11.5	N/A	0.124	N/A
Griseofulvin ^a^	14.7	79	0.4	11.5	N/A	0.155	N/A
Griseofulvin ^b^	12.8	67.5	0.3	11.5	0.121	0.158	0.209
Fenofibrate ^b^	12.8	67.5	0.4	8.93 [74]	0.100	0.148	0.214
DP1 ^b^	5.43	91.5	0.3	9.32 ± 1.2	0.068	0.138	N/A
DP3 ^b^	5.38	89.8	0.47	4.74 ± 0.32	0.065	0.120	N/A

^a^ Model C was fitted by individual run, ^b^ Model C was fitted to the complete dataset at once, and the average of the process parameters was determined.

**Figure 8:**
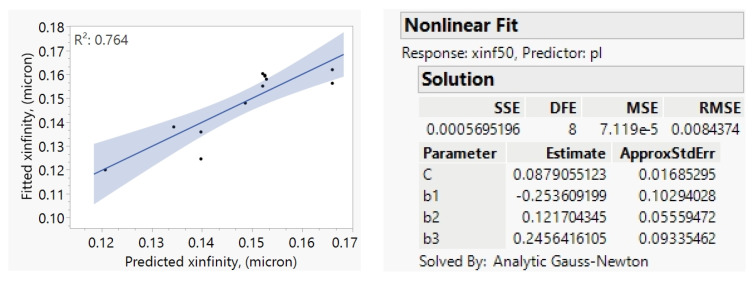
An empirical model for *x*_50,inf_ (Equation (15)) with associated parity plot and statistics for its parameters.

Following the Model C fits, we sought a power-law correlation between the fitted *x*_50,inf_ values and *U*_tip_, *BL*, *D*_b_, and *YM*_API_, but the *U*_tip_ contribution was found to be insignificant. The resulting fitted power-law correlation for *x*_50,inf_ is shown in Equation (15), with the associated fitting statistics and parity plot in Figure 8.

(15) $$x_{50,inf}=C_{50}{BL}^{b1}D_{b}^{b2}{YM}_{API}^{b3}$$

The *x*_10,inf_ and *x*_90,inf_ values were highly correlated with *x*_50,inf_. Therefore, the models for *x*_10,inf_ and *x*_90,inf_ were built by only fitting *C*_j_, the coefficient of Equation (15), and fixing the exponents *b*_1_, *b*_2_, and *b*_3_ to −0.25, 0.12 and 0.24, respectively. *C*_10_ was found to be 0.057 with *R*^2^ of 0.85, and *C*_90_ was found to be 0.125 with *R*^2^ of 1.0, since it was obtained with only two data points. The resulting equation is captured as Equation (8) in Section 3.2.

Using Equation (8), we were able to predict the apparent grinding limit (*x*_*j*,inf_) for case studies where milling time was relatively short, i.e., DP3, DP5, DP6 and DP4. When fitting models, we estimated model parameters *A*, *B*, *N* and *x*_inf_ using the complete dataset instead of obtaining model parameters for each experiment. For the sake of consistency, when estimating *x*_inf_ with Equation (8), we took the average of *BL* and *D*_b_ values if multiple bead loadings and sizes were used in the dataset, in order to obtain a single *x*_inf_ estimation that is representative of the complete dataset. These grinding limit predictions thus enabled us to use Model C (Equation (6)) with the transform in Equation (2) to perform further analysis.

A deeper examination of apparent grinding limits and Equation (8) shows that *x*_*j*,inf_ values for GF varied in a relatively narrow range as the process parameters were changed (see Table 5), and the exponents in Equation (8) were rather small, signifying no impact of *U*_tip_ (or *w*) and a weak impact of *BL* and *D*_b_. This is unsurprising because the grinding limit is mainly determined by material properties, not by the process parameters, provided the milling time is sufficiently long. In fact, the *true* grinding limit of APIs is independent of process parameters, depending solely on material properties such as *YM*_API_, hardness *H*, and fracture toughness *K*_c_, which collectively determine the brittleness of the APIs as scored by the brittleness index *BI* = *H/K*_c_, and the brittle–ductile transition size [57,58]. We speculate that the relatively low exponent of *YM*_API_ could be related to the fact that *YM*_API_ was used here as the sole descriptor of material properties due to a lack of data on other API material properties such as *H* and *K*_c_. Thus, to refine Equation (8), we would need to study a broad range of material properties, which is outside the scope of this paper.

#### 4.3.2. Mill-Scale efficiency Factor (E)

We found that most of the scaling between mills is reasonably well predicted by only considering the batch size and mill volume available for beads. Just these two terms could be used without any further consideration for mill-scale efficiency differences with average errors of approximately 20% (but could be as high as 45%) when predicting the required milling time to reach a specific particle size endpoint. In an effort to reduce this error when scaling between mills, we introduced a mill-scale efficiency factor, *E*, to score the efficiency of particular mills. The mill-scale efficiency factor is derived from parameter fitting and scores mills relative to a reference mill, here DV150. Thus, the DV150 mill-scale efficiency factor is fixed to 100%, whereas the efficiency factor for other mills is determined as a parameter fit that minimizes the sum of the squared errors between particle sizes from model and data. We can determine mill efficiency scores for each particle size quantile and drug product. We found that these efficiency scores are reasonably consistent across drug products and particle size quantiles. Mill efficiency scores may also be affected by wear and tear as the components in the mill become worn down over long time periods. We can average mill efficiency for each mill over all drug products and particle size quantile, which yields the overall mill-scale efficiency factor, *E*, for each mill. We used the *E* terms that were obtained by averaging the individual efficiencies fitted to each drug product and size class. Readers can follow the same procedure for their own mills, or they may use Equation (7) to have a rough estimate of the efficiency of their mills. The mill-scale efficiency scores for different mills and drug products are shown in Table 6. The parity plots for the efficiency fits with Model C are shown for DP1, DP2, DP3, DP4, DP5 and DP6 in Figure S3, Figure S4, Figure S5, Figure S6, Figure S7 and Figure S8, respectively.

**Figure 9:**
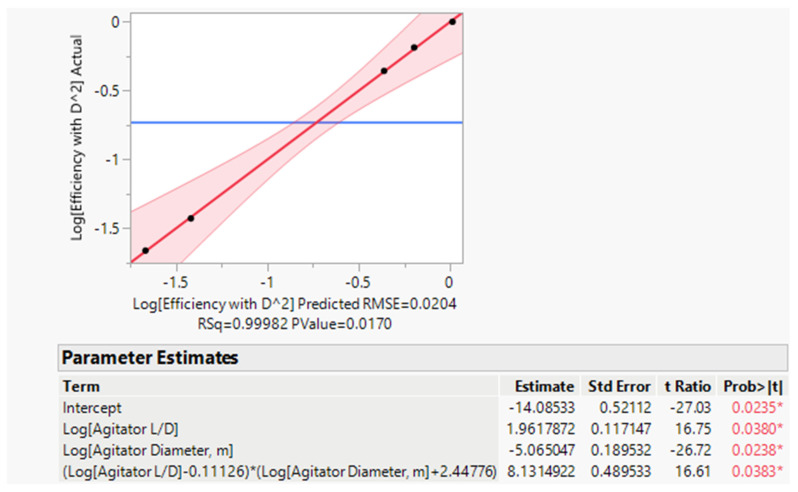
Model fit and parity plot for Equation (7): natural logarithm of the mill efficiencies as a function of logarithm of agitator diameter, agitator length to diameter ratio and their interaction. * Indicates parameter significance.

We explored how mill properties might affect the differences in the mill efficiencies and developed an empirical correlation (see Equation (7) in Section 3.2) describing mill-scale efficiency factor as a function of tip diameter and the length to diameter ratio of the agitator (blade) length to mill chamber diameter. The model fit and parity plots for Equation (7) are shown in Figure 9. If readers are interested in using the Model C with a mill with different dimensions, Equation (7) (Section 3.2.3) can be used to estimate the mill efficiency. Though the parameters have highly significant estimates in this model, we caution readers that this may be only applicable to Netzsch mills, which have many of the mill dimensions scaled to correlate with the agitator diameter. Since there are many mill dimensions that scale similarly to agitator diameter and length to diameter ratio in our study, it is not unexpected to find those parameters and their interaction to be significant predictors of mill efficiencies that could be explained with a multiple linear regression model such as the following.

#### 4.3.3. Milling Rate (*A_j_*)

In this section, we used Model C, with the mill-scale efficiency factors reported in Table 6, to fit *A_j_*, *B_j_*, *N_j_*, *K*2*_j_*, and *x*_*j*,inf_ for each case study. To generalize when to fit the *x*_*j*,inf_ term and when to predict it via Equation (8), the decision criteria were selected as fit if endpoint *x*_10_, *x*_50_, *x*_90_ < 0.1, 0.15, 0.3 μm, respectively, and predict via Equation (8) otherwise. The model fits and parity plots can be seen for FNB, GF, DP1, DP2, DP3, DP4, DP5 and DP6 in Figure S9, Figure S10, Figure S11, Figure S12, Figure S13, Figure S14, Figure S15 and Figure S16, respectively. Table 7 summarizes all the fitted model parameters, where the unit of *A*_j_ is $\frac{{\mathrm{kg}}^{0.4}}{m^{2.4}s^{1.8}}$, *B*_j_ and *N*_j_ are dimensionless, *x*_inf,j_ is in μm, and *K2*_j_ is in °K.

**Figure 10:**
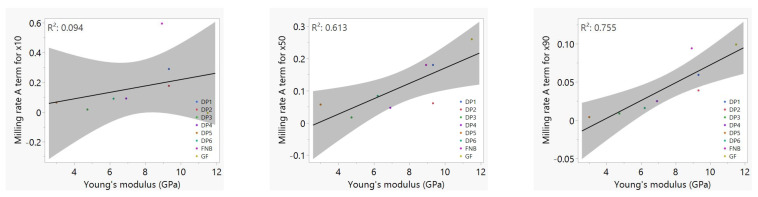
Milling rate *A* and Young’s modulus of the drugs in various case studies for *x*_10_ (**left**), *x*_50_ (**middle**), and *x*_90_ (**right**).

Similar to *x*_*j*,inf_, the parameters *A_j_*, *B_j_*, and *N_j_* can be mathematically correlated with drug material properties. We note that these parameters may also be impacted by formulation properties, but since the formulation was kept constant in each case study, and the measurements for suspension properties such as viscosity and density were not available in most case studies, we did not investigate their relationship with the model parameters in the scope of this paper. For such correlations to be accurate, the suspension must be stable in terms of particle size growth and crystallinity. In addition, having fixed exponents in Model C helps in identifying what factors impact the model parameters other than process parameters in a dataset that consists of multiple drug products. As a first attempt to correlate milling characteristics of drugs with drug properties, in Section 4.3.1, we sought correlations for *A_j_*, which had a significant fit in all studies. The correlations of the milling rate term *A_j_* with *YM*_API_ are shown in Figure 10. This correlation for *A_j_* was stronger for the coarser quantiles, as shown by the higher *R*^2^ values. Additional predictors such as the viscosity of the suspension and other material properties like *H* and *K*_c_ could strengthen the correlation. The relationships observed in Figure 10 suggest that wet bead milling processes of materials with a *YM*_API_ of less than approximately 4 GPa may be impractical for drug product development due to the predicted low milling rates of the *x*_50_ and *x*_90_. The milling rate *A_j_* parameter trends toward zero below a *YM*_API_ of 3 GPa. This tends to manifest as a bi-modal population where a large size class influencing the *x*_90_ is time consuming to eliminate. Generally, low YM materials will require lower milling temperatures, higher bead loading, and larger tip speeds to achieve desired size specifications within reasonable manufacturing times at large scales. While YM correlates with milling rate, *H* and *K*_c_ may improve this correlation and similar correlations can be sought for B and N parameters in the future.

## 5. Typical Model Use and Validation

Thus far, model development has been described without the mention of the order in which the data were gathered or how the model is typically used during product development. Once Model C was built on the first three drug products (DP1-3), the model started to be used for a priori prediction of scale-up from small scale batches. Typically, Model C uses DV50 and/or DV150 data on small volume batches to estimate the A, B and N terms, thus allowing for the prediction of processing times for large batches produced on the DV300. At times, the model has been used to prospectively predict both results of scale changes, as well as tip speed changes to reduce processing times such that a large batch can be milled in a single shift. The model has been used prospectively to predict scale-up for DP4-6. This provides an ongoing validation that the model can be used to predict milling operations in advance after being trained on very limited data, often only one or two batches at a small scale.

**Figure 11:**
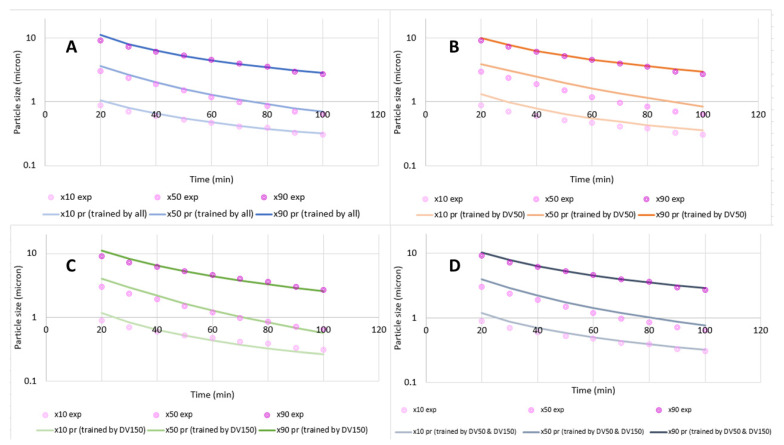
Use of DV50 and/or DV150 Data to prospectively predict DV300 outcome for DP6: (**A**) model trained by all data, (**B**) model trained by DV50 data only, (**C**) model trained by DV150 data only), and (**D**) model trained by DV50 and DV150 data.

An illustrative example for Drug Product 6 (DP6) is shown in Figure 11. These plots show experimental and predicted particle sizes versus milling time for a run at a DV300 scale. The predictions in Figure 11A are for the model in Figure S16, which was trained by all available experiments. The model was trained on DV50 and DV150 data to predict the DV300 run. The predictions in Figure 11B are for the model trained via only DV50 data. The predictions in Figure 11C are for the model trained via only DV150 data. Finally, the predictions in Figure 11D are for the model trained via DV50 and DV150 data. For these cases, the model prediction fidelity was subsequently verified based on experimental data at the DV300 scale. In all cases, the predictions are very well aligned with the experimental observations, confirming the prediction fidelity of the model.

## 6. Conclusions

In this work, we developed a semi-mechanistic modeling framework with elements from microhydrodynamic theory to predict particle size evolution in pharmaceutical wet bead milling over a wide range of process conditions and scales. Models A, B, and C, with different levels of complexity and practical utility, offer pharmaceutical engineers a wide range of capabilities to simulate, scale-up, and understand their processes. Model A, derived directly from microhydrodynamic theory, is the most general model and entails significant effort to parameterize. The full potential of Model A should be tested in a future study. Being simple and computationally convenient, Model B is expected to reduce the amount of experimentation needed to develop, optimize, scale-up, scale-down, and scale-out the wet bead milling process. We demonstrated the wide applicability of Model B in multiple case studies across drug products and mill scales. Model C, though developed by fitting to data for the specific drug products and mills described here, requires even fewer experiments to parametrize and deploy to future milling batches. We demonstrated the power of such a drug-product- and mill-agnostic model to provide insights into the most important mechanisms that govern milling rates outside of process parameters.

In the future, Model B can be further refined by considering formulations in a wider range of viscosity and flow regimes. The impact of residence time distribution may be incorporated through circuit Pe correlations as a function of tip speed, axial mean velocity of the suspensions, and number of turnovers. Future research should also consider the dependence of apparent and true grinding limit on the hardness and fracture toughness of APIs in addition to Young’s modulus. Overall, this comprehensive theoretical and experimental study has provided a semi-mechanistic modeling framework that can be adopted and adapted in a fit-for-purpose manner by pharmaceutical engineers to simulate, optimize, and scale wet bead milling processes.

## Figures and Tables

**Table 1:** Overview of wet bead mills utilized and associated experiments.

Location	GSK	GSK	GSK	GSK	GSK	NJIT
Equipment	DV50	DV150	DV300	DV2000	DV4000	MicroCer
Drug products used	Proprietary formulations					Griseofulvin, Fenofibrate
	DP5, DP4, DP6	DP3, DP2, DP4, DP6	DP3, DP1, DP5, DP2, DP6	DP1	DP1	
Batch volume (L)	0.1–0.5	0.3–1	1–5	10–30	30–200	0.2
Milling time (hour)	0.5–4	0.5–4	1–6	3–10	6–40	3
Number of rotors (-)	2	5	8	7	8	2
Chamber diameter, *D*_m_ (mm)	76	76	76	128	180	77
Agitator Diameter, *D*_a_ (mm)	68.5	65	65	110	152	60
Agitator Length, *L*_a_ (mm)	25.0	65.5	118	170	255	32
100% Bead Mill Volume, *V*_m_ (mL)	56.3	157	243	1659	4120	60
Range of Tip speed, *U*_tip_ (m/s)	4.5–6	4.5–5.5	4–7.8	4–6.6	5–6.5	11–14.7
Bead Loading, *BL* (%)	75, 85	85	75–99.8	80–90	85	56–79
Bead size, *D*_b_ (mm)	0.3, 0.65	0.3, 0.65	0.3, 0.65	0.3	0.3	0.2–0.4
# of experiments	8	4	20	8	6	18

**Table 2:** Comparison of the modeling approaches described in this work.

	Micro-Hydrodynamic Model, Model A (Section 3.2.1)	Semi-Mechanistic Model, Model B (Section 3.2.2)	Pre-Calibrated Semi-Mechanistic Model, Model C (Section 3.2.3)
Description	MHD-based mechanistic model	Flexible semi-mechanistic model	Pre-calibrated version of Model B based on particle size data from several studies
Fitting Parameters	A*, *B*_j_, *N*_j_, *x*_j,inf_, (+more parameters to develop a model for Power)	*A*_j_, *B*_j_, *N*_j_, *x*_j,inf_, *K*2*_j_*, *E*, *N*_1_, *N*_2_, *N*_3_, *N*_4_	*A*_j_, *B*_j_, *N*_j_, *K*2*_j_*, *x*_j,inf_
Complexity Level	Highest	Medium	Lowest
Number of Experiments needed	1 experiment to calibrate model parameters (after power is estimated)	Design of Experiments (DoE) varying mill scale, tip speed, bead loading, size, material, chiller set temperature	If *K*2*j* needs to be fitted 2; otherwise, only 1 experiment
When it should be preferred?	If power during milling and viscosity and density of the suspension are known	If data from a full DoE is available to calibrate this more flexible model, which would represent a specific application and parameter ranges with less error	If experimentation is costly and the materials and parameter ranges used are similar to those in this study
Advantage	Less dependency on the particle size data	Can be applied to all applications from pharmaceuticals to inorganic materials, and all parameter ranges of interest	The most efficient both experimentally and computationally
Disadvantage	Less predictive capability as it depends on experimental input for power. To have the same capability as Model B and C, a power model should be developed	High risk of overfitting; experimentally costly	Application outside the ranges used in this study is not evaluated

**Table 3:** Fitted exponents of normalized *ω* (*N*_1_) in various case studies.

Study	*x*_10_ Rotor Speed Exponent	*x*_50_ Rotor Speed Exponent	*x*_90_ Rotor Speed Exponent
DP1 2000 mL Scale (Section 4.2.1)	1.71	1.84	1.64
DP1 All Scales (Section 4.2.1)	2.05	1.62	1.39
NJIT Bead Size Study (Supplementary Materials)	N/A	1.99	N/A
NJIT Bead Type Study (Supplementary Materials)	N/A	2.13	2.02

**Table 4:** Fitted exponent of BL (*N*_2_) averaged across *x*_10_, *x*_50_ and *x*_90_ quantiles in various case studies.

Study	Bead Loading, *BL*	Fitted Exponent, *N*_2_	Relative Milling Rate Due to Bead Loading, *BL^N^*_2_, Observed
DP3	99.80%	1.29	0.769
DP1 DV2000	90%	2.89	0.737
DP1 DV2000	85%	2.89	0.625
DP1 DV2000	80%	2.89	0.525
NJIT Bead Size	79%	2.84	0.512
NJIT Bead Size	68%	2.84	0.334
NJIT Bead Size	56%	2.84	0.193
NJIT Bead Type	79%	3.24	0.466
NJIT Bead Type	56%	3.24	0.153

**Table 6:** Summary of the fitted efficiencies.

Study	Size Class	DP1	DP2	DP3	DP4	DP5	DP6	Average ± Std	Mill-Scale Efficiency Factor, *E*
DV50	*x*_10_	N/A	N/A	N/A	0.85	0.68	0.50	0.68 ± 0.18	0.70
	*x*_50_	N/A	N/A	N/A	0.83	0.69	0.55	0.69 ± 0.14	
	*x*_90_	N/A	N/A	N/A	0.82	0.73	0.64	0.73 ± 0.09	
DV150	*x*_10_	N/A	1	1	1	N/A	1	1	1.0
	*x*_50_	N/A	1	1	1	N/A	1	1	
	*x*_90_	N/A	1	1	1	N/A	1	1	
DV300	*x*_10_	0.83	0.89	0.78	N/A	1.1	0.74	0.87 ± 0.14	0.83
	*x*_50_	0.83	0.79	0.68	N/A	1.2	0.82	0.86 ± 0.20	
	*x*_90_	0.83	0.75	0.30	N/A	1.1	0.80	0.76 ± 0.29	
DV2000	*x*_10_	0.24	N/A	N/A	N/A	N/A	N/A	0.23	0.24
	*x*_50_	0.23	N/A	N/A	N/A	N/A	N/A	0.23	
	*x*_90_	0.25	N/A	N/A	N/A	N/A	N/A	0.25	
DV4000	*x*_10_	0.16	N/A	N/A	N/A	N/A	N/A	0.15	0.19
	*x*_50_	0.19	N/A	N/A	N/A	N/A	N/A	0.17	
	*x*_90_	0.28	N/A	N/A	N/A	N/A	N/A	0.25	

**Table 7:** Model C fitted parameters for all drug products.

Drug Name	GF	FNB	DP1 ^a^	DP2	DP3 ^a^	DP4	DP5	DP6
*A*_10_	−0.006	0.594	0.289	0.176	0.017	0.091	0.064	0.090
*B*_10_	1.00	1.98 × 10^−3^	0.122	0.011	0.570	0.026	0.038	0.063
*N*_10_	−0.014	1.52	0.350	0.192	0.207	1.08	0.852	0.812
*x*_10,inf_	0.121	0.100	0.068	N/A	0.065	N/A	N/A	N/A
*K*2_10_	N/A	N/A	N/A	N/A	N/A	N/A	−3236	N/A
*A*_50_	0.260	0.180	0.180	0.061	0.017	0.047	0.057	0.084
*B*_50_	0.183	0.025	0.120	0.447	0.416	0.006	0.144	0.159
*N*_50_	0.632	0.927	0.330	0.211	0.262	1.22	0.486	0.450
*x*_50,inf_	0.158	0.148	0.138	N/A	0.119	N/A	N/A	N/A
*K*2_50_	N/A	N/A	−1412	N/A	N/A	N/A	−2160	N/A
*A*_90_	0.099	0.094	5.93 × 10^−2^	0.039	8.82 × 10^−3^	0.025	4.14 × 10^−3^	0.016
*B*_90_	0.340	0.140	1.63 × 10^−3^	0.350	0.344	0.001	0.001	0.004
*N*_90_	0.340	0.536	0.666	0.211	0.270	1.19	1.38	0.989
x_90,inf_	0.210	0.214	N/A	N/A	N/A	N/A	N/A	N/A
*K*2_90_	N/A	N/A	−5842	N/A	N/A	N/A	−4175	N/A

^a^ Equation (11) was used in the model.

## Data Availability

The data are contained within this article and its Supplementary Materials.

## Supplementary Materials

The following supporting information can be downloaded at: https://www.mdpi.com/article/10.3390/pharmaceutics16030394/s1, Figure S1. Parity plots and model fits for fenofibrate drug product/Microcer dataset for *1/x10*, *1/x50*, *1/x90* from Model B (Equation (5)) with *N1, N2*, and *N3* as fitted parameters; Figure S2. Parity plots and model fits for griseofulvin drug product/Microcer dataset for *1/x10*, *1/x50*, *1/x90* from Model B (Equation (5)) with *N1*, *N2*, and *N4* as fitted parameters; Figure S3. Mill-scale efficiency factor parity plots and fits for DV300 DV2000 and DV4000 for DP1; Figure S1. Mill-scale efficiency factor parity plots and fits for DV300 for DP2; Figure S2. Mill-scale efficiency factor parity plots and fits for DV300 for DP3.; Figure S3. Mill-scale efficiency factor parity plots and fits for DV50 for DP4; Figure S4. Mill-scale efficiency factor parity plots and fits for DV50 and DV300 for DP6; Figure S5. Mill-scale efficiency factor parity plots and fits for DV300 for DP5; Figure S6. Parity plots and model fits for NJIT bead material case study in Section 4.2.2 (fenofibrate); Figure S7. Parity plots and model fits for NJIT bead size case study in Section 4.2.3 (griseofulvin); Figure S8. Parity plots and model fits for DP1, using Model C (Equation (6)); Figure S9. Parity plots and model fits for DP2 using Model C (Equation (6); Figure S10. Parity plots and model fits for DP3 using Model C (Equation (6)); Figure S11. Parity plots and model fits for DP4 using Model C (Equation (6)); Figure S12. Parity plots and model fit for DP5 using Model C (Equation (6)); Figure S13. Parity plots and model fits for DP6 using Model C (Equation (6)). Figure S14. Parity plots and model fits for DP4 using Model C (Equation (10)). Figure S15. Parity plots and model fit for DP5 using Model C (Equation (10)). Figure S16. Parity plots and model fits for DP6 using Model C (Equation (10)).

## Nomenclature

Symbols used	
*A_j_*	Slope term of the Yt function in Model B and C for each particle size quantile j (10, 50, 90), kg^0.4^/(m^2.4^s^1.8^)
*A_j_**, *A_j_***	Intermediately derived slope terms of Yt function for each particle size quantile j (10, 50, 90)
*a*	The average frequency of drug particle compressions, 1/min
API	Active pharmaceutical ingredient
*B_j_*	Intercept term of the Yt function for each particle size quantile j (10, 50, 90)
*BI*	Brittleness index, m^−1/2^
*BL*	Bead loading, % *v*/*v*
*c*	Fractional volumetric bead loading in the drug suspension–beads mixture
*C*	Rate constant
CPS	Crosslinked polystyrene
*D*	Diameter, m
*e*	Restitution coefficient
*E*	Mill-scale efficiency correction factor
*g*_0_	Radial distribution function at contact
*h*	Thickness of the powder bed, m
*H*	Hardness, Pa
*k*	Apparent breakage rate constant, 1/min
*K*_c_	Fracture toughness, Pa.m^1/2^
Kt	Arrhenius equation for the temperature impact
*m*	Mass, kg
MHD	Microhydrodynamic model
*N_j_*	Shape factor of the Yt transform for each particle size quantile j (10, 50, 90)
*N*_1_	Exponent for the stirrer speed effect
*N*_2_	Exponent for the bead loading effect
*N*_3_	Exponent for the bead density effect
*N*_4_	Exponent for the bead size effect
*N*_5_	Exponent for the tip diameter effect
*N*_6_	Exponent for the suspension viscosity effect
*N*_7_	Exponent for the suspension density effect
*N*_t_	Number of turnovers
*P*	Power, W
*P*_ax_	Axial pressure, Pa
*P*_rad_	Radial pressure, Pa
*P*_v_	Power density, W/m^3^
Pe	Peclet number
*PR*	Poisson ratio
PSD	Particle size distribution
*Q*	Volumetric flow rate or pumping rate of the suspension, m^3^/s
*R*	Radius, m
*R*_diss_	Effective drag coefficient
Re	Reynolds number
*S*	Specific breakage rate, 1/min
*t*	Milling time, min
*T*	Temperature, °C
*U*_tip_	Tip speed, m/s
*V*	Volume, mL
*x_j_*	Particle size for each particle size quantile j (10, 50, 90), m
*YM*	Young’s modulus, Pa
Yt	Transform of the dependent variable Y
YSZ	Yttrium-stabilized zirconia
Greek letters	
*ε*	Powder compact out-of-die porosity
*ε*_coll_	Energy dissipation rate due to partially inelastic bead–bead collisions, W/m^3^
*ε*_ht_	Power spent on shear of milled suspension of the slurry at the same shear rate but calculated (measured) when no beads were present in the flow, W/m^3^
*ε*_m_	Nondimensional bead–bead gap thickness at which the lubrication force stops increasing and becomes a constant, –
*ε*_visc_	Energy dissipation rate due to both the liquid–beads viscous friction and lubrication, W/m^3^
*ε*	powder compact out-of-die porosity
*γ*	Mass concentration, g/mL
λ	Lumped parameters of the microhydrodynamic model
*θ*	Granular temperature, m^2^/s^2^
*μ*	Viscosity, Pa.s
*ρ*	Density, kg/m^3^
σ_ax_	Axial stress, Pa
σ_rad_	Radial stress, Pa
*τ*	Mean residence time for the single pass, min
*ω*	Rotational speed of the rotor, 1/min
Indices	
10	10% passing size of the cumulative PSD
50	Median particle size of the cumulative PSD
90	90% passing size of the cumulative PSD
a	agitator
batch	Batch
b	Bead
c	Out-of-die compacts
inf	Infinity
*j*	Index for particle size quantile
lim	Grinding limit
m	Mill chamber
p	Particle
ref	Reference values used to make variables nondimensional
s	Suspension

## Appendix A. A Theoretical Basis for Linearizing Data Transformations

In Section 3.1, we have described the particle size data transformation employed for data linearization. We derived Equations (1) and (2) from a purely breakage kinetics perspective, providing a theoretical basis for the statistical transforms. The timewise evolution of particle size quantile *x_j_* is described by the following empirical *n*^th^-order breakage kinetics model (see, e.g., Guner et al. [48] for the change in the median size *x*_50_ without the grinding limit):

(A1) $$dx_{j}/dt=-S_{j}x_{j}^{n_{j}}\;\;with\;t=0\;\;x_{j}\left(0\right)=x_{j,ini}$$

where *S_j_* is the specific breakage rate function and $x_{j,ini}$ is the initial particle size at *t* = 0. Here, *S_j_* is particle size *x_j_* dependent. In view of the linear *x*_p_ dependence of *a* in Equation (3), we assumed a functional form *S_j_* = *C_j_x_j_* and integrated Equation (A1) after separation of variables. Here, *C_j_* is a breakage rate constant. After some algebraic manipulations, this resulted in the following:

(A2) $${\left(\frac{1}{x_{j}}\right)}^{n_{j}}={n_{j}C}_{j}t+{\left(\frac{1}{x_{j,ini}}\right)}^{n_{j}}$$

This can be re-written in the form of Equation (1), recognizing that *N_j_* = *n_j_*, *k_j_* = *n_j_C_j_*, and ${\left(\frac{1}{x_{j,ini}}\right)}^{n_{j}}=B_{j}$. Let us now consider milling with a grinding limit *x*_*j*,inf_. After inserting *S_j_* = *C_j_*(*x_j_*–*x*_*j*,inf_) for *x_j_* ≥ *x*_*j*,inf_ and *S_j_* = 0 for *x_j_* < *x*_*j*,inf_ into the following kinetic model by Guner et al. [48]:

(A3) $$dx_{j}/dt=-S_{j}{\left(x_{j}-x_{j,inf}\right)}^{n_{j}}\;\;with\;t=0\;\;x_{j}\left(0\right)=x_{j,ini}$$

and integrating the resultant differential equation following separation of variables, we obtained the following expression after some algebraic manipulations:

(A4) $${\left(\frac{1/x_{j}}{1/x_{j,inf}-1/x_{j}}\right)}^{n_{j}}={n_{j}C}_{j}{x_{j,inf}}^{n_{j}}t+{\left(\frac{x_{j,lim}}{x_{j,ini}-x_{j,lim}}\right)}^{n_{j}}$$

This can be re-written in the form of Equation (2), recognizing that *N_j_* = *n_j_*, *k_j_* = *n_j_C_j_x_j_*_,inf_*^nj^* and ${\left(\frac{x_{j,lim}}{x_{j,ini}-x_{j,lim}}\right)}^{n_{j}}=B_{j}$. Equation (1) is supported by two different mechanistic modeling approaches. First, an analytical solution of the PBM for batch milling, which is identical to that for continuous plug-flow milling, can be used to predict the variation in the mean particle size *x*_m_ = *C/t*^1/*N*^, for sufficiently long milling times [54,75]. Here, the constant *C* depends on the parameters of the specific breakage rate function *S* and self-similar breakage distribution function, and *N* is the power-law exponent of the size-dependent *S*. This relation can be rearranged to give a linear relation in *t*: (1/*x*_m_)*^N^* = *C*^−*N*^*t*. As the self-similar solution gives *x_j_* = *k_j_*^*^*x*_m_, we find linear relations in time for *x_j_*, i.e., (1/*x_j_*)*^N^* = *k_j_t*, where $k_{j}={\left(k_{j}^{*}C\right)}^{N}$, and arrive at the theoretical origin of Equation (1) with *N_j_* = *N*. In a completely different avenue of theoretical development, based on a microhydrodynamic model along with the Charles’ energy–average particle size relationship [76], Eskin et al. [59] described the evolution of particle size as a function of time *t*, which can also be reduced to the form (1/*x_j_*)*^N^* = *k_j_t* for long milling times.

## Appendix B. Microhydrodynamic Theory for Wet Bead Milling

In Section 3.2.1, we described the microhydrodynamics-based wet bead milling model. We presented the key equations of the microhydrodynamic theory for wet bead milling; all assumptions and derivation steps can be found in [29]. The power (or its density *P*_v_) inside a wet stirred mill is spent via three energy dissipation mechanisms as mathematically expressed as follows:

(A5) $$P_{v}=\varepsilon_{visc}+\varepsilon_{coll}+\varepsilon_{ht}$$

Here, *ε*_visc_ is the energy dissipation rate due to both the liquid–beads viscous friction and lubrication; *ε*_coll_ is the energy dissipation rate due to partially inelastic bead–bead collisions; and *ε*_ht_ accounts for the power spent on shearing the slurry at the same shear rate without the beads. Inserting the respective expressions for *ε*_visc_ and *ε*_coll_, Equation (A5) turns into the following:

(A6) $$P_{v}=\frac{54\mu_{s}c\theta R_{diss}}{D_{b}^{2}}+\frac{12}{D_{b}\sqrt{\pi }}\left(1-e^{2}\right)g_{0}c^{2}\rho_{b}\theta^{3/2}+\varepsilon_{ht}$$

where *θ* is the granular temperature defined as one third of the bead–milled suspension relative mean-square velocity, *R*_diss_ is the effective drag coefficient, *e* is the restitution coefficient for the bead–bead collisions, and *g*_0_ is the radial distribution function at contact. In a more comprehensive microhydrodynamic model developed by Guner et al. [29], the following Lun model [77] was used for *g*_0_:

(A7) $$g_{0}={\left[1-{\left(c/c_{lim}\right)}^{1/3}\right]}^{-1}$$

as it exhibited a better predictive capability of the microhydrodynamics and breakage kinetics. In Equation (A7), *c*_lim_ is the packing limit and equal to 0.63 [78]. Wylie et al. [79] give *R*_diss_ as follows:

(A8) $$R_{diss}=R_{diss0}+KD_{b}\rho_{s}\theta^{0.5}/\mu_{s}$$

where *K* is a coefficient given by an empirical correlation of bead concentration *c*:

(A9) $$K=(0.096+0.142c^{0.212})/{\left(1-c\right)}^{4.454}$$

*R*_diss0_ in Equation (A8) is the dissipation coefficient considering squeezing of the milled suspension film between two approaching beads, and it is expressed as follows:

(A10) $$R_{diss0}=k_{1}-k_{2}\log\varepsilon_{m}$$

In Equation (A10), *ε*_m_ is the nondimensional bead–bead gap thickness at which the lubrication force stops increasing and becomes a constant. Parameters *k*_1_ and *k*_2_ were computed using the following:

(A11) $$k_{1}=1+3\sqrt{c/2}+\left(135/64\right)c\log c+11.26c(1-5.1c+16.57c^{2}-21.77c^{3})$$

(A12) $$k_{2}=cg_{0}$$

All parameters and variables in Equation (A6) are either known or experimentally measured except for the granular temperature, which can be easily solved by any nonlinear equation solver. Next, granular temperature can be used to calculate the average frequency of drug particle compressions between the beads *a*, which is the multiplication of the probability of single drug particle to be caught by the beads *p* and the average oscillation frequency of a single bead ν.

(A13) $$a=p\nu =23.28\frac{c^{2}{\left(1-{\left(\frac{c}{c_{lim}}\right)}^{0.33}\right)}^{-1}}{\sqrt{\pi }(1-c)}{\left[\frac{\rho_{b}\left(1-{PR}_{b}^{2}\right)}{{YM}_{b}}\right]}^{0.4}{\frac{x_{p}}{D_{b}^{2}}\theta }^{0.9}$$

To derive a rough scaling approximation for *ω* and *D*_a_ dependence of *θ* when only ω or *D*_a_ is varied to affect *P*_v_, keeping all process–formulation–design parameters fixed, we neglected *ε*_ht_, defined *z*^2^ = *θ*, and rewrote Equation (A6) as a cubic equation of the form, *P*_v_ − *λ*_1_*z*^2^ *−* (*λ*_2_+*λ*_3_)*z*^3^ = 0, where *λ*_1_, *λ*_2_, and *λ*_3_ correspond to the constant positive-valued factors obtained from plugging Equation (A8) into Equation (A6). Instead of finding the only real, positive-valued root analytically, for which a numerical solution is much more convenient as mentioned above and as implemented in previous microhydrodynamic studies [29,60], we performed an asymptotic analysis: First, *λ*_1_ << *λ*_2_ + *λ*_3_ was assumed, which corresponds to turbulent flow with negligible viscosity effects (formulations with low viscosity) and yields an approximate solution: *θ* ≅ *P*_v_^2/3^/(*λ*_2_ + *λ*_3_)^2/3^. The other asymptotic solution pertains to viscous formulations for which the viscous effects tend to dominate (laminar flow), i.e., *λ*_1_ >> *λ*_2_ + *λ*_3_. In this case, we found *θ* ≅ *P*_v_/*λ*_1_. The exponent of *P*_v_ is theoretically bound between 2/3 and 1. Note that these two asymptotic solutions were not intended to replace either a numerical solution or a full analytical solution; rather, in the absence of any other information, it allowed us to estimate the *w* and *D*_a_ dependence of *θ* under the limiting condition of turbulent flow (see Section 3.2).

## Appendix C. Derivation of Model B from Model A

In Section 3.2.2, we described the development of a semi-mechanistic model for wet bead milling. The connectivity between Equations (3) and (5) is explained here. The particle size term in Equation (A13) has been wrapped in the slope term in Equation (4). As the time dependence of *x*_p_ was already modeled by the transformation *Y*t in Equations (1) and (2), for the sake of convenience and preservation of dimensional homogeneity, we set *x*_p_ = *x_j_*(0) = *x_j_*_,ini_. A similar approach was also adopted to assess the impacts of various process parameters and bead properties in earlier microhydrodynamic studies (e.g., [29,48]). Plugging Equation (A13) into Equation (3) and lumping all constants into a new parameter $A_{j}^{**}=0.02328x_{j,ini}A_{j}^{*}/\sqrt{\pi }$, we obtained Equation (4) of the main text (Model A). To formulate a microhydrodynamically inspired semi-mechanistic model capable of scale-up, we assumed that *θ* could be described empirically as a power-law model as follows:

(A14) $$\frac{\theta }{\theta_{ref}}={\left(\frac{\omega }{\omega_{ref}}\right)}^{y_{1}}{\left(\frac{c}{c_{ref}}\right)}^{y_{2}}{\left(\frac{\rho_{b}}{\rho_{b,ref}}\right)}^{y_{3}}{\left(\frac{D_{b}}{D_{b,ref}}\right)}^{y_{4}}{\left(\frac{D_{a}}{D_{a,ref}}\right)}^{y_{5}}{\left(\frac{\mu_{s}}{\mu_{s,ref}}\right)}^{y_{6}}{\left(\frac{\rho_{s}}{\rho_{s,ref}}\right)}^{y_{7}}{Kt}_{j}^{y_{8}}E^{y_{9}}$$

where ref stands for reference mill and bead conditions and *D*_a_ is the agitator’s tip (blade) diameter. Here, *E* is a mill scale factor that accounts for the energy transfer efficiency of any mill with respect to a reference mill, which captures the impact of mill design differences at different scales; it is constant for a given mill and independent of the API formulation. The DV150 mill was chosen as the reference mill since it was the most commonly used mill in the investigated studies. Kt is a factor that accounts for temperature change from a reference temperature (15 °C), which is an Arrhenius function with a *K*2*_j_* parameter to be fitted $\exp\left[-{K2}_{j}\left(\frac{1}{273.15+\left(T\;℃\right)}-\frac{1}{288.15}\right)\right]$. Thus, the $\mu_{s}$ and *ρ*_s_ terms account for the impact of any formulation change on the viscosity and density at the reference temperature, whereas Kt accounts for all temperature-induced changes to the viscosity for a given formulation.

Before substituting Equation (A14) into Equation (4), we made some simplifying assumptions by setting *y*_7_ = *y*_8_ = 10/9, which resulted in Yt*_j_* changing linearly with *E* and *K*t*_j_*. The reference bead density *ρ*_b,ref_ was set to that of Yttrium-stabilized zirconia (YSZ) beads *ρ*_YSZ_. We also defined *A_j_* = ${C^{*}A}_{j}^{**}{\theta_{ref}}^{0.9}{\rho_{YSZ}}^{0.4}/\left(D_{a,ref}^{N_{5}}c_{lim}^{0.9y_{2}}D_{b.ref}^{2}\right)$ and *BL = c/c*_lim_. After substituting Equation (A14) into Equation (4) and performing some algebraic manipulations, we arrived at Equation (5) of the main text (Model B) with exponents *N*_1_–*N*_7_, the pre-factor *A_j_*, and the intercept *B_j_*. For a given drug formulation, the viscosity and suspension density terms are set to 1. If temperature effects are neglected or for nearly isothermal operation, ${Kt}_{j}$ is set to 1. The one-to-one relationships between the exponents of the ${Yt}_{j}$ model in Equation (5) and the *θ* model in Equation (A14) were as follows: $N_{1}=0.9y_{1}$, $N_{3}=0.9y_{3}+0.4$, $N_{4}=2-0.9y_{4}$, $N_{5}=0.9y_{5}$, $N_{6}=0.9y_{6}$, and $N_{7}=0.9y_{7}$. *N*_2_ was approximated by simplifying $c^{2+0.9*y2}/\left[1-{\left(c/c_{lim}\right)}^{0.33}\right]$ as $C^{*}{\left(c/c_{lim}\right)}^{N_{2}}=C^{*}{BL}^{N_{2}}$, where *C*^*^ is a fitting constant.
