# Supplementary material for: Development of a Semi-Mechanistic Modeling Framework for Wet Bead Milling of Pharmaceutical Nanosuspensions
Source: Pharmaceutics. 2024 Mar 13;16(3):394. doi: 10.3390/pharmaceutics16030394 (PMC10974034; doi:10.3390/pharmaceutics16030394)
Supplement: Supplementary file 1 [file pharmaceutics-16-00394-s001.zip › pharmaceutics-2905975-supplementary.pdf]

Article

SUPPLEMENTARY MATERIALS

Development of a Semi-Mechanistic Modeling Framework for Wet Bead Milling of Pharmaceutical Nanosuspensions

Donald J. Clancy [1,*,†], Gulenay Guner [1,2,†], Sayantan Chattoraj [1], Helen Yao [1], M. Connor Faith [1], Zahra Salahshoor [1], Kailey N. Martin [1] and Ecevit Bilgili [2]

[1] GlaxoSmithKline R&D, Collegeville, PA 19426, USA
[2] Otto H. York Department of Chemical and Materials Engineering, New Jersey Institute of Technology, Newark, NJ 07102, USA

* Correspondence: donald.j.clancy@gsk.com
† Primary authors contributed equally to this work.

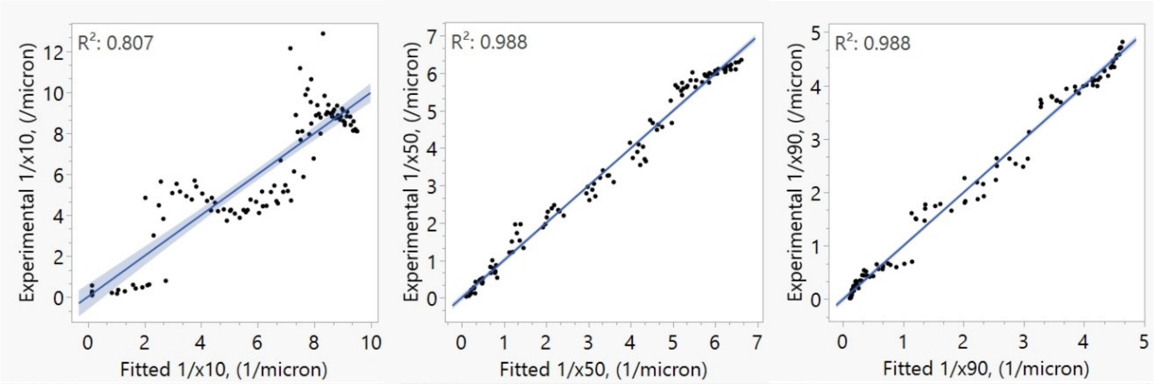

Nonlinear Fit

Response: 1/x10, Predictor: Yt x10 N1 N2 N3 fits

Solution

SSE	DFE	MSE	RMSE
222.90704931	104	2.143337	1.464014

Parameter	Estimate	ApproxStdErr
A	0.0846652845	0.09528419
B	0.0022574806	0.01224699
N	1.4571452873	0.23063866
xinf	0.1024489709	0.00602789
N1	4.1686380321	0.87258558
N2	4.6523412403	0.71024138
N3	1.2925202556	0.1414655

Solved By: Analytic Gauss-Newton

Nonlinear Fit

Response: 1/x50, Predictor: Yt x50 N1 N2 N3 fits

Solution

SSE	DFE	MSE	RMSE
7.2192885375	106	0.0681065	0.2609722

Parameter	Estimate	ApproxStdErr
A	0.1924221355	0.03958722
B	0.0206679226	0.01213741
N	0.9419342581	0.03008724
xinf	0.1476714648	.
N1	2.1264247069	0.15854804
N2	3.371963007	0.12909678
N3	1.4590993833	0.02603035

Solved By: Analytic Gauss-Newton

Nonlinear Fit

Response: 1/x90, Predictor: Yt x90 N1 N2 N3 fits

Solution

SSE	DFE	MSE	RMSE
3.7984448803	105	0.0361757	0.190199

Parameter	Estimate	ApproxStdErr
A	0.0962051356	0.01609011
B	0.1389410105	0.03885009
N	0.5371634068	0.04410997
xinf	0.2145446514	0.00330076
N1	2.0194455805	0.12672837
N2	3.0977393648	0.10352944
N3	1.4000086614	0.02078084

Solved By: Analytic Gauss-Newton

Figure S1. Parity plots and model fits for fenofibrate drug product / Microcer dataset for $1/x_{10}$, $1/x_{50}$, $1/x_{90}$ from Model B (Eq. (5) with N1, N2, and N3 as fitted parameters (see Section 4.2.2) (Note: for x_{10} quantile, A fit is not significant).

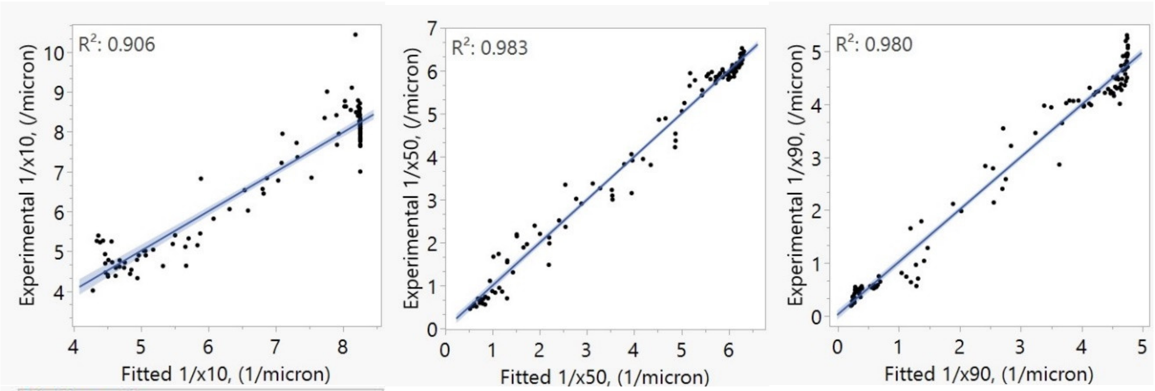

Nonlinear Fit

Response: 1/x10, Predictor: Yt x10 N1 N2 N3 fits

Solution

SSE	DFE	MSE	RMSE
27.148161965	108	0.2513719	0.50137

Parameter	Estimate	ApproxStdErr
A	-0.006496176	0.08126756
B	0.9996826176	0.00488717
N	-0.014875097	0.18877367
xinf	0.1212520344	0.00105751
N1	1.8286827465	0.45174131
N2	2.646881369	0.36315021
N4	0.1568940927	0.1673239

Solved By: Analytic Gauss-Newton

Nonlinear Fit

Response: 1/x50, Predictor: Yt x50 N1 N2 N3 fits

Solution

SSE	DFE	MSE	RMSE
9.0592271634	108	0.0838817	0.2896234

Parameter	Estimate	ApproxStdErr
A	0.2442595856	0.0658433
B	0.1870301838	0.05485835
N	0.6285921207	0.06346684
xinf	0.1577469017	0.00184524
N1	1.9949581213	0.19744494
N2	2.844936268	0.15886876
N4	0.2816692918	0.07331114

Solved By: Analytic Gauss-Newton

Nonlinear Fit

Response: 1/x90, Predictor: Yt x90 N1 N2 N3 fits

Solution

SSE	DFE	MSE	RMSE
8.3013638399	108	0.0768645	0.2772444

Parameter	Estimate	ApproxStdErr
A	0.0717311459	0.0210021
B	0.3651074183	0.0904266
N	0.318869218	0.05789181
xinf	0.2103188644	0.0026837
N1	2.0052285636	0.19044435
N2	2.2835727753	0.15430876
N4	0.0915011266	0.07143465

Solved By: Analytic Gauss-Newton

Figure S2. Parity plots and model fits for griseofulvin drug product / Microcer dataset for $1/x_{10}$, $1/x_{50}$, $1/x_{90}$ from Model B (Eq. (5) with N_1, N_2, and N_4 as fitted parameters (see Section 4.2.3). (Note: for x_{10} quantile, A and N_4 fit is not significant, for x_{90} quantile, N_4 fit was not significant.)

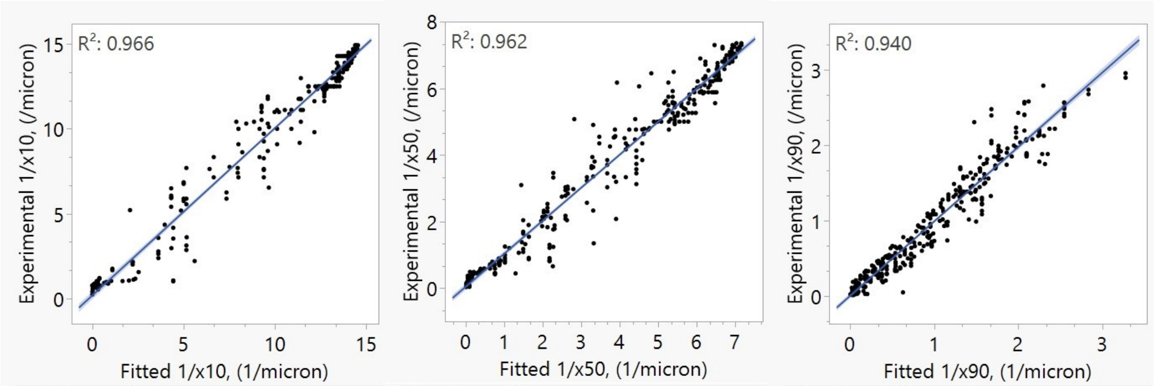

Nonlinear Fit

Response: 1/x10, Predictor: Yt x10 E match

Solution

SSE	DFE	MSE	RMSE
257.89713612	299	0.8625322	0.9287261

Parameter	Estimate	ApproxStdErr
A	0.3616308273	0.04011525
B	0.0207890395	0.10623003
N	0.3515128788	0.04404879
xinf	0.0685445127	0.00070997
E2000	0.2338399373	0.0082202
E4000	0.1487568701	0.00332919
K2	-4083.53059	1748.091

Solved By: Analytic Gauss-Newton

Nonlinear Fit

Response: 1/x50, Predictor: Yt x50 E match

Solution

SSE	DFE	MSE	RMSE
79.838930608	336	0.2376159	0.4874586

Parameter	Estimate	ApproxStdErr
A	0.1887811242	0.0220486
B	0.1409683222	0.10289327
N	0.3169430734	0.04767609
xinf	0.1384370092	0.00250577
E2000	0.2298798263	0.00497528
E4000	0.1739708561	0.00361491
K2	-1687.078906	657.992424

Solved By: Analytic Gauss-Newton

Nonlinear Fit

Response: 1/x90, Predictor: Yt x90 E match

Solution

SSE	DFE	MSE	RMSE
11.707516722	338	0.0346376	0.1861119

Parameter	Estimate	ApproxStdErr
A	0.0564884678	0.0008541
B	0.0280321556	.
N	0.6026562415	0.01201184
xinf	0.1911912311	.
E2000	0.246899337	0.00532896
E4000	0.2500965416	0.0047204
K2	-5773.309303	682.480776

Solved By: Analytic Gauss-Newton

Figure S3. Mill scale efficiency factor parity plots and fits for DV300 DV2000 and DV4000 for DP1.

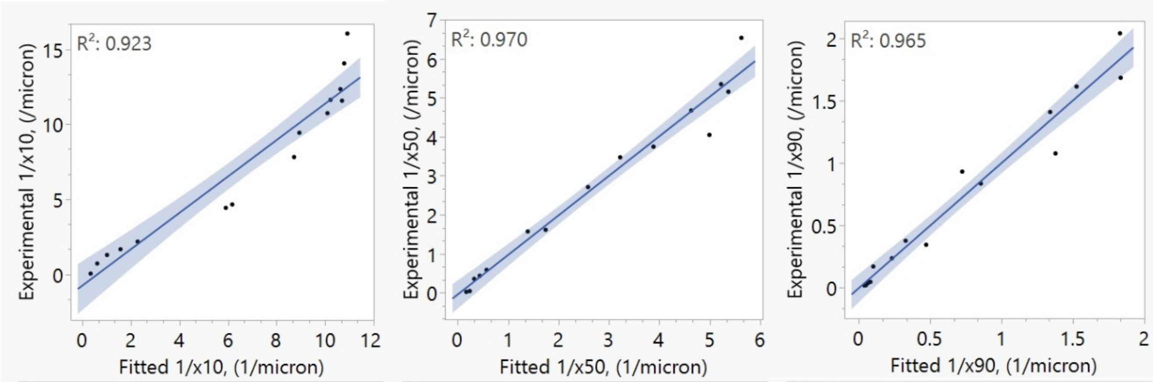

Nonlinear Fit

Response: 1/x10, Predictor: Yt x10 E match

Solution

SSE	DFE	MSE	RMSE
49.03977695	13	3.7722905	1.9422385

Parameter	Estimate	ApproxStdErr
A	0.1000745865	0.01945259
B	0.5	.
N	0.2	.
xinf	0.0887976793	.
E300	0.8882433028	0.23280651

Solved By: Analytic Gauss-Newton
Identifiability issues involving at least: B N xinf

Nonlinear Fit

Response: 1/x50, Predictor: Yt x50 E match

Solution

SSE	DFE	MSE	RMSE
2.0047578032	11	0.1822507	0.4269083

Parameter	Estimate	ApproxStdErr
A	0.0571605748	0.03985007
B	0.4949763904	0.34219674
N	0.189123028	0.14314232
xinf	0.1370911541	.
E300	0.7932700182	0.05205747

Solved By: Analytic Gauss-Newton
Identifiability issues involving at least: xinf

Nonlinear Fit

Response: 1/x90, Predictor: Yt x90 E match

Solution

SSE	DFE	MSE	RMSE
0.2396463357	11	0.021786	0.1476009

Parameter	Estimate	ApproxStdErr
A	0.0370793401	0.03182314
B	0.4403956777	0.50727505
N	0.1663074977	0.19289547
xinf	0.1947317529	.
E300	0.7482256243	0.02914578

Solved By: Analytic Gauss-Newton
Identifiability issues involving at least: xinf

Figure S4. Mill scale efficiency factor parity plots and fits for DV300 for DP2. Note: Since the datasets are missing timepoints from the first 2 hours of milling, the intercept term could not be predicted significantly and fixed.

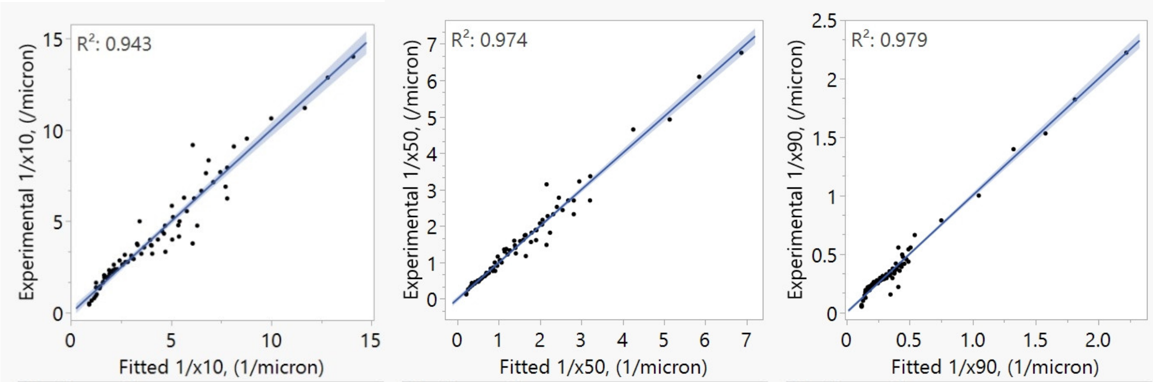

Nonlinear Fit

Response: 1/x10, Predictor: Yt x10 E match

Solution

SSE	DFE	MSE	RMSE
38.974900526	74	0.5266878	0.7257326

Parameter	Estimate	ApproxStdErr
A	0.0210438536	0.00790529
B	0.4958820534	0.22325382
N	0.2536112286	0.14033054
xinf	0.0651898693	0.00664245
E300	0.7825245909	0.0509335
K2	1e-100	.

Solved By: Analytic Gauss-Newton
Identifiability issues involving at least: K2

Nonlinear Fit

Response: 1/x50, Predictor: Yt x50 E match

Solution

SSE	DFE	MSE	RMSE
3.3602209179	74	0.0454084	0.2130924

Parameter	Estimate	ApproxStdErr
A	0.0239363028	0.00225464
B	0.1611417462	0.09102259
N	0.4876897368	0.10868604
xinf	0.108802791	0.01433265
E300	0.6847138314	0.03235394
K2	1e-100	.

Solved By: Analytic Gauss-Newton
Identifiability issues involving at least: K2

Nonlinear Fit

Response: 1/x90, Predictor: Yt x90 E match

Solution

SSE	DFE	MSE	RMSE
0.2093607008	75	0.0027915	0.0528344

Parameter	Estimate	ApproxStdErr
A	0.0095954167	0.0003931
B	0.0343299962	0.00893239
N	0.8714649541	0.05561763
xinf	0.1708329678	.
E300	0.3023864005	0.02314572
K2	1e-100	.

Solved By: Analytic Gauss-Newton
Identifiability issues involving at least: xinf K2

Figure S5. Mill scale efficiency factor parity plots and fits for DV300 for DP3. Eq .(11) was also used for the bead loading term as part of this fitting exercise.

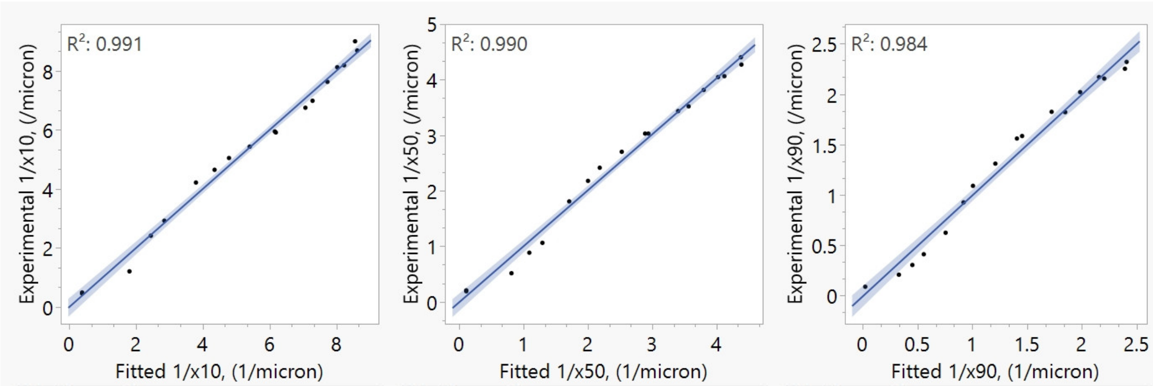

Nonlinear Fit

Response: 1/x10, Predictor: Yt x10 E match

Solution

SSE	DFE	MSE	RMSE
1.2465364589	14	0.0890383	0.2983929

Parameter	Estimate	ApproxStdErr
A	0.0821990665	0.00422988
B	0.0257602933	0.01789958
N	1.0759873839	0.05976037
xinf	0.0826736781	.
E50	0.8516594317	0.05351902

Solved By: Analytic Gauss-Newton
Identifiability issues involving at least: xinf

Nonlinear Fit

Response: 1/x50, Predictor: Yt x50 E match

Solution

SSE	DFE	MSE	RMSE
0.3641149554	14	0.0260082	0.1612706

Parameter	Estimate	ApproxStdErr
A	0.0434514761	0.00205347
B	0.0056394177	0.00783016
N	1.2130163191	0.06129734
xinf	0.1276365557	.
E50	0.8319741836	0.04911176

Solved By: Analytic Gauss-Newton
Identifiability issues involving at least: xinf

Nonlinear Fit

Response: 1/x90, Predictor: Yt x90 E match

Solution

SSE	DFE	MSE	RMSE
0.1841514858	14	0.0131537	0.1146895

Parameter	Estimate	ApproxStdErr
A	0.0236463746	0.00166512
B	0.0015473574	0.00652643
N	1.1829412723	0.07558669
xinf	0.1813019257	.
E50	0.8169243366	0.05412926

Solved By: Analytic Gauss-Newton
Identifiability issues involving at least: xinf

Figure S6. Mill scale efficiency factor parity plots and fits for DV50 for DP4.

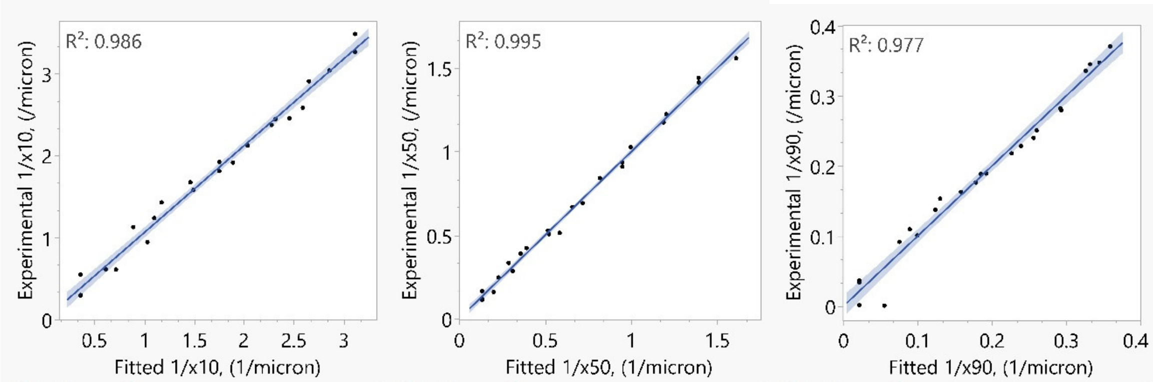

Nonlinear Fit

Response: 1/x10, Predictor: Yt x10 E match

Solution

SSE	DFE	MSE	RMSE
0.2857965942	18	0.0158776	0.1260063

Parameter	Estimate	ApproxStdErr
A	0.1115242195	0.00593167
B	0.0869331884	0.03650645
N	0.728889117	0.09682161
xinf	0.0877765609	.
E50	0.5027806468	0.02408851
E300	0.7375920856	0.0294586

Solved By: Analytic Gauss-Newton
Identifiability issues involving at least: xinf

Nonlinear Fit

Response: 1/x50, Predictor: Yt x50 E match

Solution

SSE	DFE	MSE	RMSE
0.023526805	18	0.001307	0.0361531

Parameter	Estimate	ApproxStdErr
A	0.0911081476	0.00398455
B	0.243962057	0.06541037
N	0.3523271726	0.05855857
xinf	0.1355146905	.
E50	0.5491940394	0.01237911
E300	0.8203046934	0.014866

Solved By: Analytic Gauss-Newton
Identifiability issues involving at least: xinf

Nonlinear Fit

Response: 1/x90, Predictor: Yt x90 E match

Solution

SSE	DFE	MSE	RMSE
0.0063942122	18	0.0003552	0.0188477

Parameter	Estimate	ApproxStdErr
A	0.0182192396	0.00503306
B	0.0051348002	0.00503594
N	0.960665631	0.1129407
xinf	0.1924924581	.
E50	0.6366357318	0.0372419
E300	0.8037204944	0.04305751

Solved By: Analytic Gauss-Newton
Identifiability issues involving at least: xinf

Figure S7. Mill scale efficiency factor parity plots and fits for DV50 and DV300 for DP6.

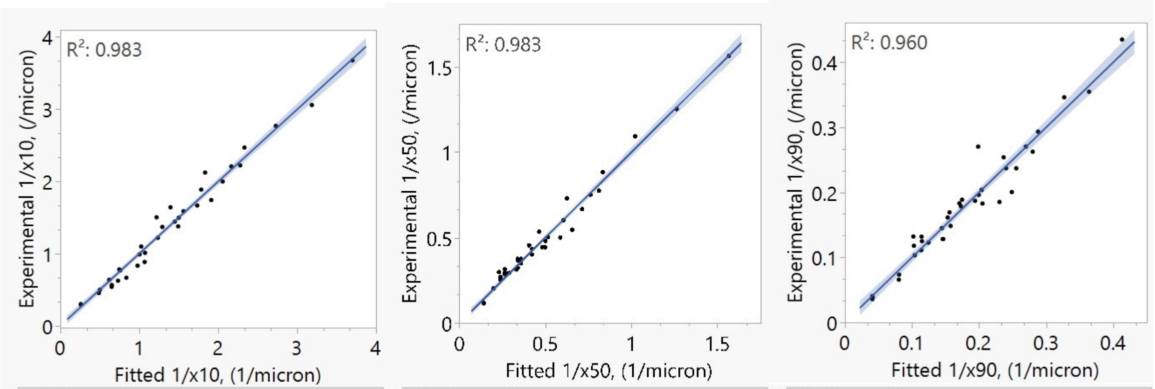

Nonlinear Fit

Response: 1/x10, Predictor: Yt x10 E match

Solution

SSE	DFE	MSE	RMSE
0.4979398633	38	0.0131037	0.1144713

Parameter	Estimate	ApproxStdErr
A	0.0763352043	0.00424632
B	0.0407414429	0.01225568
N	0.8145621555	0.05504451
xinf	0.0746067553	.
E50	0.68	.
E300	1.1352677707	0.06241188
K2	0	.

Solved By: Analytic Gauss-Newton
Identifiability issues involving at least: xinf E300

Nonlinear Fit

Response: 1/x50, Predictor: Yt x50 E match

Solution

SSE	DFE	MSE	RMSE
0.0710274629	38	0.0018691	0.0432336

Parameter	Estimate	ApproxStdErr
A	0.0610039193	0.00245087
B	0.1248804657	0.03296644
N	0.5059448768	0.0563642
xinf	0.1305519944	.
E50	0.69	.
E300	1.1521416773	0.07091346
K2	0	.

Solved By: Analytic Gauss-Newton
Identifiability issues involving at least: xinf E300

Nonlinear Fit

Response: 1/x90, Predictor: Yt x90 E match

Solution

SSE	DFE	MSE	RMSE
0.015710853	38	0.0004134	0.0203333

Parameter	Estimate	ApproxStdErr
A	0.005063291	0.00154881
B	0.0013244514	0.00088952
N	1.3188407908	0.10712261
xinf	0.1614218886	.
E50	0.73	.
E300	1.1495561162	0.10671862
K2	0	.

Solved By: Analytic Gauss-Newton
Identifiability issues involving at least: xinf E300

Figure S8. Mill scale efficiency factor parity plots and fits for DV300 for DP5, with DV50 efficiency assumed to be the average of the previously fitted values in **Figures S6** and **S7** for DP5.

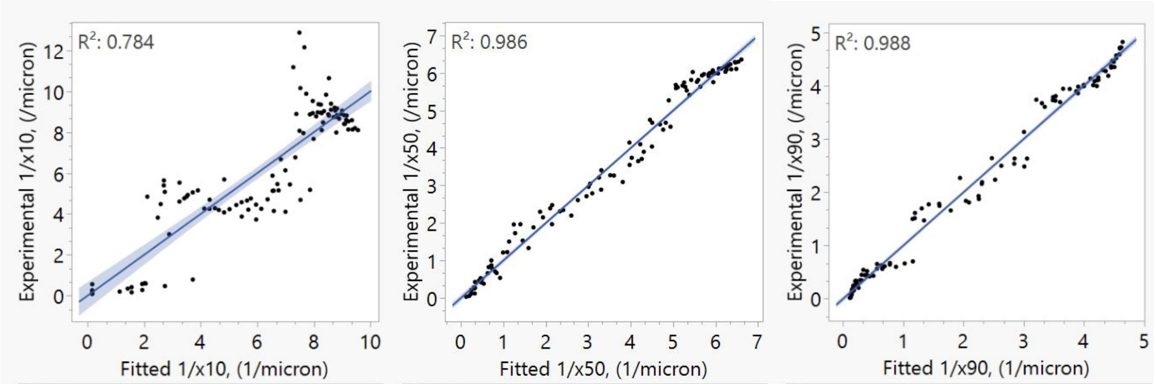

Nonlinear Fit

Response: 1/x10, Predictor: Yt 10

Solution

SSE	DFE	MSE	RMSE
248.62847398	107	2.3236306	1.524346

Parameter	Estimate	ApproxStdErr
A	0.5943668052	0.1878961
B	0.001983296	0.01065824
N	1.5170407324	0.26630855
xinf	0.0998149094	0.00764354
K2	1e-100	.

Solved By: Analytic Gauss-Newton
Identifiability issues involving at least: K2

Nonlinear Fit

Response: 1/x50, Predictor: Yt 50

Solution

SSE	DFE	MSE	RMSE
8.1499750734	109	0.0747704	0.2734418

Parameter	Estimate	ApproxStdErr
A	0.1796268411	0.00602045
B	0.0255452297	0.01331692
N	0.9274234903	0.03153093
xinf	0.1476714648	.
K2	1e-100	.

Solved By: Analytic Gauss-Newton
Identifiability issues involving at least: xinf K2

Nonlinear Fit

Response: 1/x90, Predictor: Yt 90

Solution

SSE	DFE	MSE	RMSE
3.8316748669	108	0.0354785	0.1883573

Parameter	Estimate	ApproxStdErr
A	0.0943152957	0.0044658
B	0.139638826	0.03858332
N	0.535803828	0.04366392
xinf	0.2144168393	0.00328723
K2	1e-100	.

Solved By: Analytic Gauss-Newton
Identifiability issues involving at least: K2

Figure S9. Parity plots and model fits for NJIT bead material case study in section 4.2.2 (fenofibrate), assuming mill scale efficiency factor is 100%, using Model C (Eq. (10)).

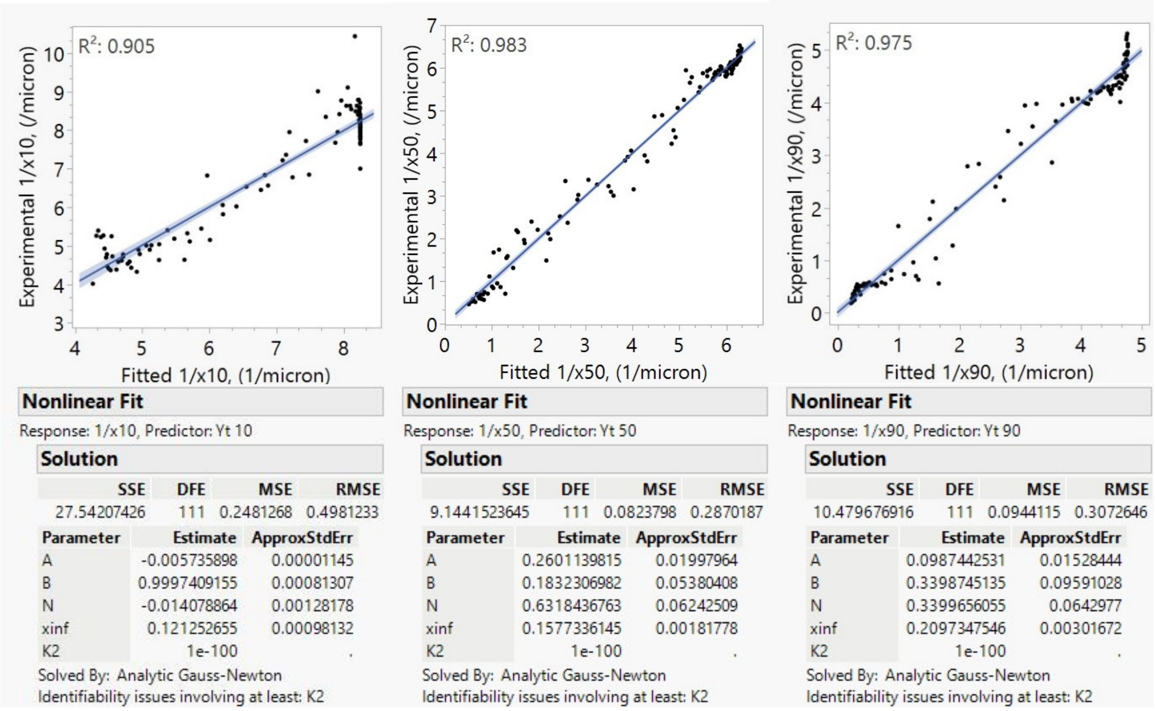

Figure S10. Parity plots and model fits for NJIT bead size case study in section 4.2.3 (griseofulvin), assuming mill efficiency is 100%, using Model C (Eq. (10)).

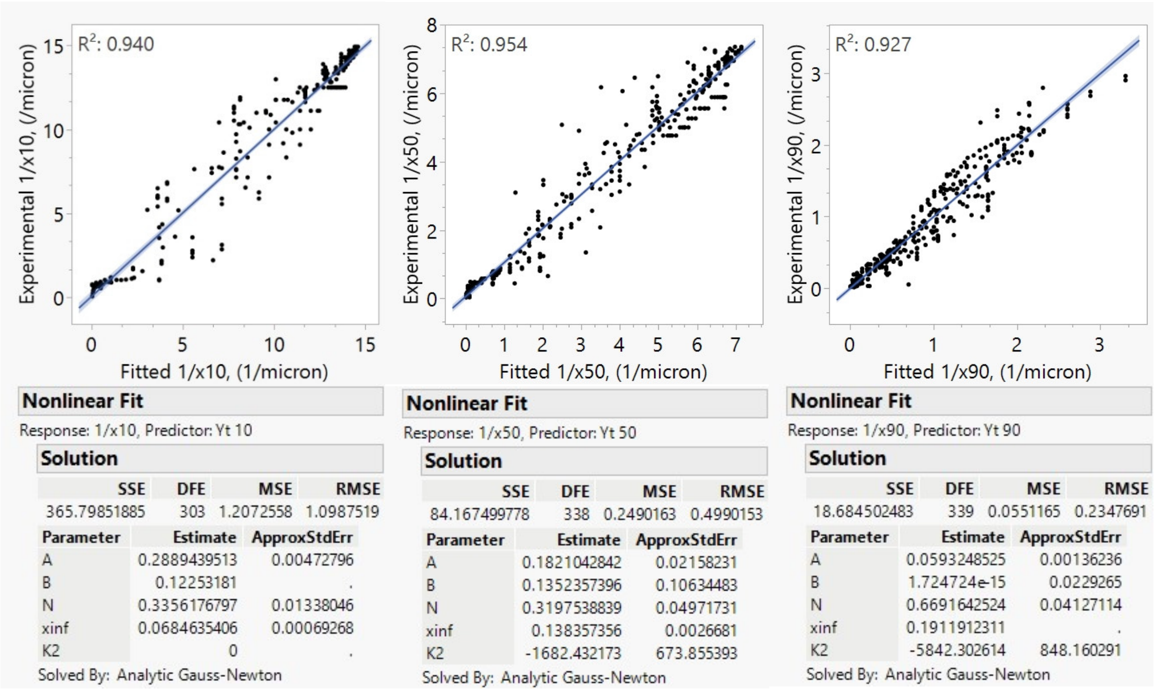

Figure S11. Parity plots and model fits for DP1, using Model C (Eq. (10)).

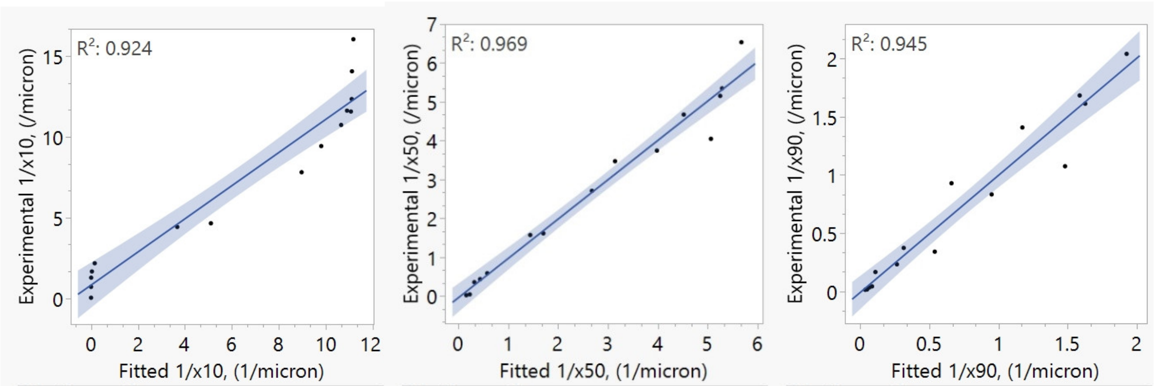

Nonlinear Fit

Response: 1/x10, Predictor: Yt 10

Solution

SSE	DFE	MSE	RMSE
46.154379188	13	3.5503369	1.8842338

Parameter	Estimate	ApproxStdErr
A	0.176832027	0.01488624
B	0.0109460712	.
N	0.1922783391	0.07825986
xinf	0.0887976793	.
K2	1e-100	.

Solved By: Analytic Gauss-Newton
Identifiability issues involving at least: A xinf K2

Nonlinear Fit

Response: 1/x50, Predictor: Yt 50

Solution

SSE	DFE	MSE	RMSE
2.087506986	12	0.1739589	0.4170838

Parameter	Estimate	ApproxStdErr
A	0.0611197628	0.03670238
B	0.4474786828	0.31947767
N	0.2109173179	0.13796316
xinf	0.1370911541	.
K2	1e-100	.

Solved By: Analytic Gauss-Newton
Identifiability issues involving at least: xinf K2

Nonlinear Fit

Response: 1/x90, Predictor: Yt 90

Solution

SSE	DFE	MSE	RMSE
0.3804161842	12	0.0317013	0.1780487

Parameter	Estimate	ApproxStdErr
A	0.0392413959	0.02827186
B	0.3499051392	0.50880473
N	0.211171681	0.22722735
xinf	0.1947317529	.
K2	1e-100	.

Solved By: Analytic Gauss-Newton
Identifiability issues involving at least: xinf K2

Figure S12. Parity plots and model fits for DP2 using Model C (Eq. (10)).

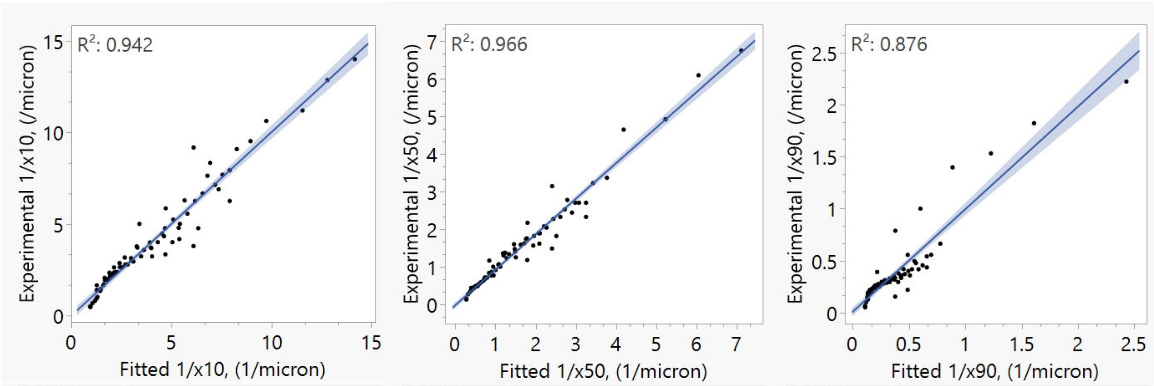

Nonlinear Fit

Response: 1/x10, Predictor: Yt 10

Solution

SSE	DFE	MSE	RMSE
39.391809726	75	0.5252241	0.7247235

Parameter	Estimate	ApproxStdErr
A	0.017176712	0.00779385
B	0.5695034993	0.23346088
N	0.2069688005	0.13281957
xinf	0.0651894614	0.00658509
K2	1e-100	.

Solved By: Analytic Gauss-Newton
Identifiability issues involving at least: K2

Nonlinear Fit

Response: 1/x50, Predictor: Yt 50

Solution

SSE	DFE	MSE	RMSE
4.2362309752	75	0.0564831	0.2376617

Parameter	Estimate	ApproxStdErr
A	0.0171511058	0.00318389
B	0.4162646025	0.16732461
N	0.2615987329	0.1003245
xinf	0.1198097093	0.01384229
K2	1e-100	.

Solved By: Analytic Gauss-Newton
Identifiability issues involving at least: K2

Nonlinear Fit

Response: 1/x90, Predictor: Yt 90

Solution

SSE	DFE	MSE	RMSE
1.2639190627	76	0.0166305	0.1289594

Parameter	Estimate	ApproxStdErr
A	0.0088240812	0.00134107
B	0.34433306	0.11985925
N	0.2701368831	0.07705088
xinf	0.1708329678	.
K2	1e-100	.

Solved By: Analytic Gauss-Newton
Identifiability issues involving at least: xinf K2

Figure S13. Parity plots and model fits for DP3 using Model C (Eq. (10)). Here, Eq. (11) was used for the bead loading term since there were experiments with >95% bead loading.

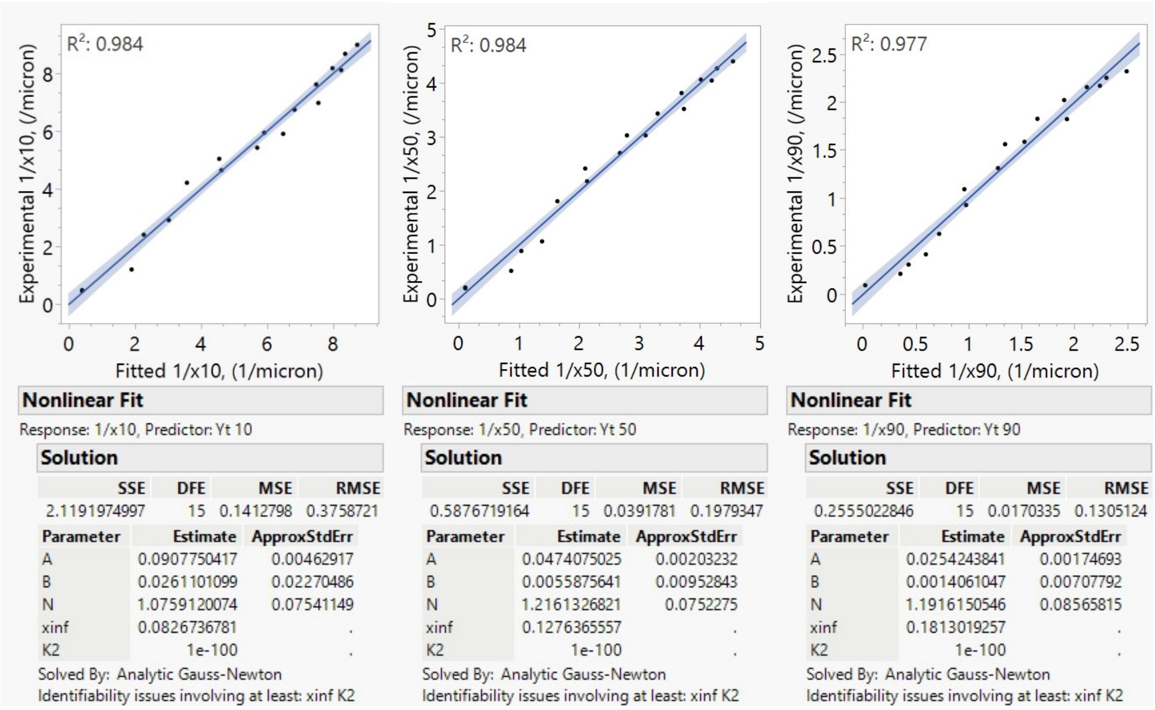

SSE	DFE	MSE	RMSE
2.1191974997	15	0.1412798	0.3758721

Parameter	Estimate	ApproxStdErr
A	0.0907750417	0.00462917
B	0.0261101099	0.02270486
N	1.0759120074	0.07541149
xinf	0.0826736781	.
K2	1e-100	.

Solved By: Analytic Gauss-Newton
Identifiability issues involving at least: xinf K2

SSE	DFE	MSE	RMSE
0.5876719164	15	0.0391781	0.1979347

Parameter	Estimate	ApproxStdErr
A	0.0474075025	0.00203232
B	0.0055875641	0.00952843
N	1.2161326821	0.0752275
xinf	0.1276365557	.
K2	1e-100	.

Solved By: Analytic Gauss-Newton
Identifiability issues involving at least: xinf K2

SSE	DFE	MSE	RMSE
0.2555022846	15	0.0170335	0.1305124

Parameter	Estimate	ApproxStdErr
A	0.0254243841	0.00174693
B	0.0014061047	0.00707792
N	1.1916150546	0.08565815
xinf	0.1813019257	.
K2	1e-100	.

Solved By: Analytic Gauss-Newton
Identifiability issues involving at least: xinf K2

Figure S14. Parity plots and model fits for DP4 using Model C (Eq. (10)).

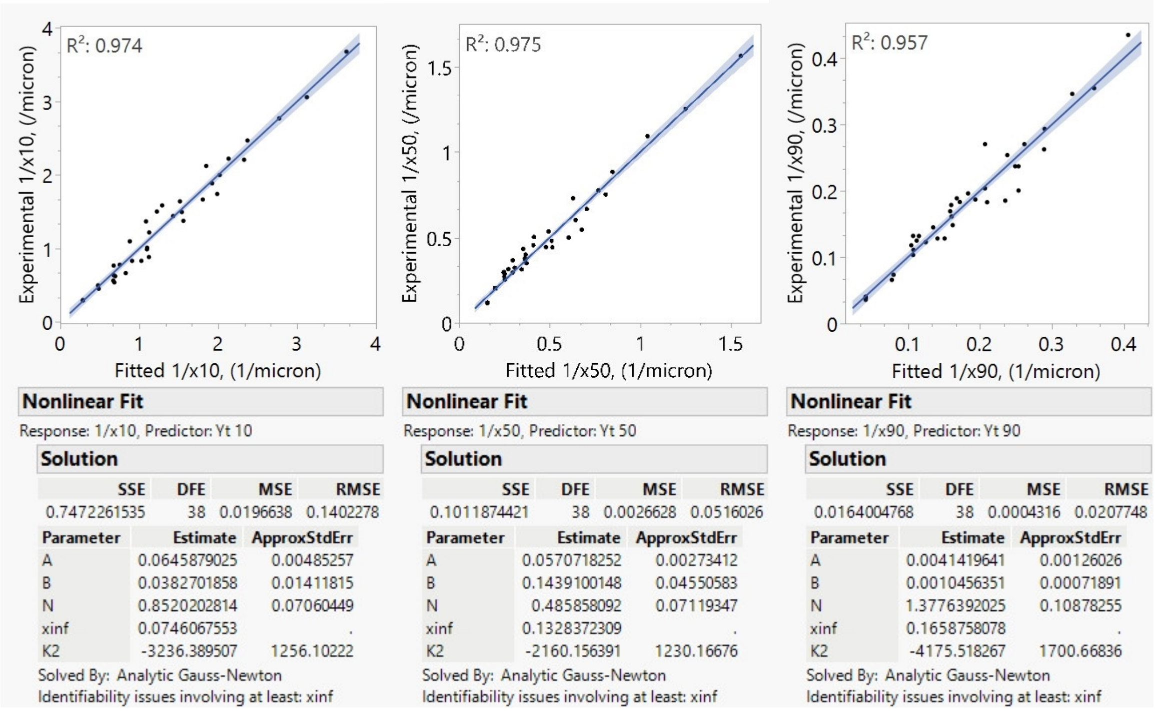

SSE	DFE	MSE	RMSE
0.7472261535	38	0.0196638	0.1402278

Parameter	Estimate	ApproxStdErr
A	0.0645879025	0.00485257
B	0.0382701858	0.01411815
N	0.8520202814	0.07060449
xinf	0.0746067553	.
K2	-3236.389507	1256.10222

Solved By: Analytic Gauss-Newton
Identifiability issues involving at least: xinf

SSE	DFE	MSE	RMSE
0.1011874421	38	0.0026628	0.0516026

Parameter	Estimate	ApproxStdErr
A	0.0570718252	0.00273412
B	0.1439100148	0.04550583
N	0.485858092	0.07119347
xinf	0.1328372309	.
K2	-2160.156391	1230.16676

Solved By: Analytic Gauss-Newton
Identifiability issues involving at least: xinf

SSE	DFE	MSE	RMSE
0.0164004768	38	0.0004316	0.0207748

Parameter	Estimate	ApproxStdErr
A	0.0041419641	0.00126026
B	0.0010456351	0.00071891
N	1.3776392025	0.10878255
xinf	0.1658758078	.
K2	-4175.518267	1700.66836

Solved By: Analytic Gauss-Newton
Identifiability issues involving at least: xinf

Figure S15. Parity plots and model fit for DP5 using Model C (Eq. (10)).

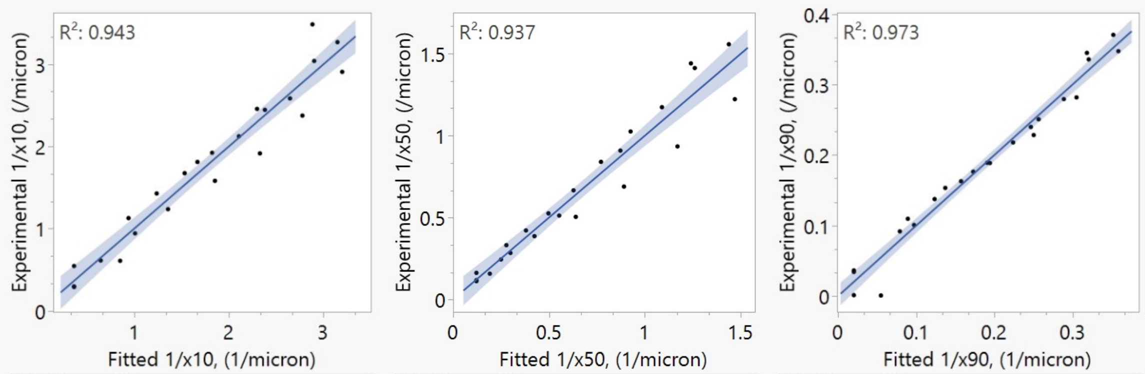

Nonlinear Fit

Response: 1/x10, Predictor: Yt 10

Solution

SSE	DFE	MSE	RMSE
1.1581702413	20	0.0579085	0.2406419

Parameter	Estimate	ApproxStdErr
A	0.0905518814	0.01083387
B	0.0627461476	0.05331561
N	0.8120037205	0.18455841
xinf	0.0877765609	.
K2	1e-100	.

Solved By: Analytic Gauss-Newton
Identifiability issues involving at least: xinf K2

Nonlinear Fit

Response: 1/x50, Predictor: Yt 50

Solution

SSE	DFE	MSE	RMSE
0.2893837064	20	0.0144692	0.1202879

Parameter	Estimate	ApproxStdErr
A	0.0845646044	0.00577916
B	0.1586483287	0.15407206
N	0.4500301167	0.19722932
xinf	0.1355146905	.
K2	1e-100	.

Solved By: Analytic Gauss-Newton
Identifiability issues involving at least: xinf K2

Nonlinear Fit

Response: 1/x90, Predictor: Yt 90

Solution

SSE	DFE	MSE	RMSE
0.0074425283	20	0.0003721	0.0192906

Parameter	Estimate	ApproxStdErr
A	0.0161972421	0.00449917
B	0.0041500732	0.0042907
N	0.989366161	0.11332748
xinf	0.1924924581	.
K2	1e-100	.

Solved By: Analytic Gauss-Newton
Identifiability issues involving at least: xinf K2

Figure S16. Parity plots and model fits for DP6 using Model C (Eq. (10)).
